# Structural and Functional Characterization of *Drosophila melanogaster* α-Amylase

**DOI:** 10.3390/molecules28145327

**Published:** 2023-07-11

**Authors:** Moez Rhimi, Jean-Luc Da Lage, Richard Haser, Georges Feller, Nushin Aghajari

**Affiliations:** 1 Molecular Microbiology and Structural Biochemistry, UMR5086, CNRS, University of Lyon 1, 7 Passage du Vercors, F-69367 Lyon, CEDEX 07, France; moez.rhimi@inrae.fr (M.R.);; 2 Evolution, Génomes, Comportement, Ecologie, UMR 9191 University Paris-Saclay—CNRS—IRD, F-91190 Gif-sur-Yvette, France; jean-luc.da-lage@universite-paris-saclay.fr; 3 Laboratory of Biochemistry, Center for Protein Engineering-InBioS, Institute of Chemistry B6a, University of Liège, B-4000 Liège, Belgium; gfeller@uliege.be

**Keywords:** α-amylase, glycoside hydrolase family 13 (GH13), crystal structure, acarbose, inhibitors, *Drosophila melanogaster*, adaptation, calcium binding, flexible loop, evolution

## Abstract

Insects rely on carbohydrates such as starch and glycogen as an energy supply for growth of larvae and for longevity. In this sense α-amylases have essential roles under extreme conditions, e.g., during nutritional or temperature stress, thereby contributing to survival of the insect. This makes them interesting targets for combating insect pests. *Drosophila melanogaster* α-amylase, DMA, which belongs to the glycoside hydrolase family 13, sub family 15, has been studied from an evolutionary, biochemical, and structural point of view. Our studies revealed that the DMA enzyme is active over a broad temperature and pH range, which is in agreement with the fluctuating environmental changes with which the insect is confronted. Crystal structures disclosed a new nearly fully solvated metal ion, only coordinated to the protein via Gln263. This residue is only conserved in the subgroup of *D. melanogaster* and may thus contribute to the enzyme adaptive response to large temperature variations. Studies of the effect of plant inhibitors and the pseudo-tetrasaccharide inhibitor acarbose on DMA activity, allowed us to underline the important role of the so-called flexible loop on activity/inhibition, but also to suggest that the inhibition modes of the wheat inhibitors WI-1 and WI-3 on DMA, are likely different.

## 1. Introduction

Carbohydrates such as starch, glycogen and other oligo- and polysaccharides provide an important energy source for insect larval growth and adult longevity preservation [1]. The resulting energy value is tightly related to the enzymes required for the bioconversion of the polysaccharides to monosaccharides that are suitable for uptake from the larval gut [2]. For insects the main reported enzymes acting on α-1,4-glucans are α-amylases and α-glucosidases [3], which have been identified in larval midguts from Hymenoptera, Orthoptera, Hemiptera, Lepidoptera, Coleoptera and Diptera [3,4]. As in plants, fungi and bacteria, α-amylases often form multigene families in animals. The multicopy nature, which allows various levels of amplification and sequence or regulation divergence, remains the rule. Some species, however, have a single gene as seen for example in the honeybee. A few species have completely lost their *Amy* gene, e.g., the louse *Pediculus humanus* and the aphid *Acyrthosiphon pisum* [4], perhaps linked to their parasitic nature. Gene copies may undergo more or less dramatic divergence, such as in *Drosophila ananassae* [5].

**Figure 1:**
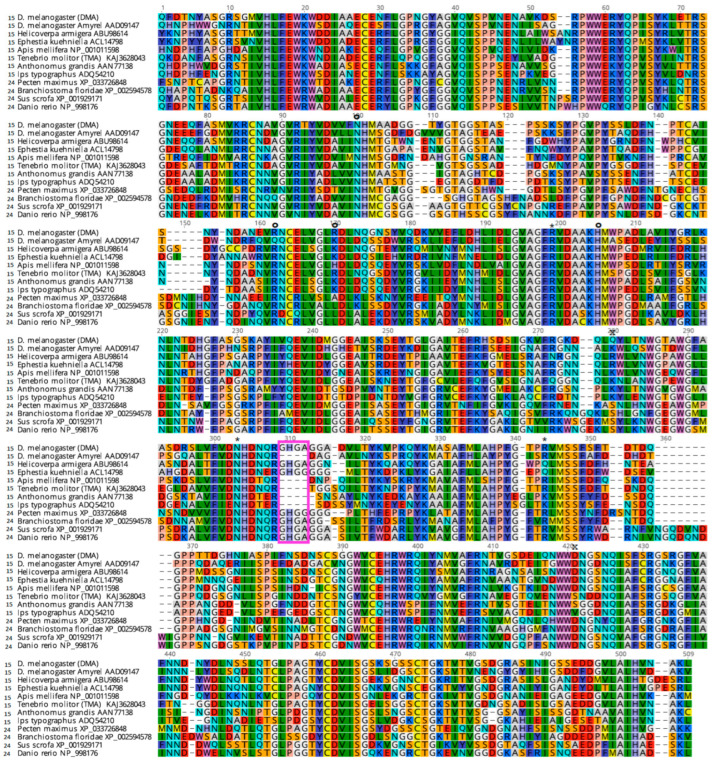
Alignment of mature α-amylase protein sequences of various animals, from subfamilies GH13_15 marked as “15” and GH13_24 marked as “24”. Amino acids are colored according to chemical properties. Circles indicate Ca^2+^/metal binding sites, asterisks indicate Cl^−^ binding sites. The cross « x » indicates the additional Ca^2+^/metal binding site found in these studies. The position of the flexible loop is boxed in pink.

It has been shown that α-amylases play an essential role in insect survival under temperature or nutritional stress [2]. Thus, these enzymes constitute attractive targets for combating insect pests [6]. Indeed, plant produced inhibitors are an important element of the plant defense response to insect predation against feeding [7]. α-Amylases (α-1,4-glucan-4-glucanohydrolase, EC 3.2.1.1) belong to the glycoside hydrolase family 13 (GH13) as classified in the CAZy database [8], and catalyze the hydrolysis of starch and related α-1,4-linked glycosyl polysaccharides [9,10]. The mature enzymes display 470–500 amino acids and share 50–55% sequence identity across animals (see Figure 1 for illustration). Some conserved sequence motifs typical of animal α-amylases have been described [11], and point to functionally important parts of the enzyme. In mosquitoes, tissue-specific differentiation (salivary vs. midgut) was observed, along with striking sequence divergence and the recruitment of an N-terminal extra sequence [12,13]. In true flies, it can be mentioned that *Drosophila melanogaster* Amy and its paralog Amyrel (“amylase related”) have diverged and display 61% sequence identity and 75% sequence similarity [4], which has led to striking differences in the activities of these enzymes [14,15].

Convergent α-amylase sequence variations observed in unrelated species suggest that selective pressures act upon these regions for better adaptation to the environment, including temperature and food availability, and deleterious metabolites. Interestingly, this is the case for the so-called “flexible loop”, which has been frequently deleted or shortened in a number of sequences (boxed in pink in Figure 1). Since this loop has been shown to be involved in a trap–release mechanism [16], its presence in *Drosophila melanogaster* α-amylase, henceforth DMA, or its absence as in *Tenebrio molitor* larvae α-amylase (TMA) could have an impact, amongst others, on the interaction with inhibitors from food [17]. Historically, α-amylases were amongst the first proteins for which molecular polymorphism was studied [18]. In the debate on selection vs. neutral evolution, it was discussed whether electrophoretic variants within a species displayed differences in their biochemical properties (i.e., some variants would be favored under given conditions) or whether they were enzymatically equivalent (i.e., neutral). *Drosophila* was a preferred model in these studies, due to the natural variability of *D. melanogaster* [19,20,21].

α-Amylases have been extensively studied from a biochemical/physical and structural biology point of view, and much is known about the relationships between their structure, function and activity (see, e.g., [22,23,24,25,26,27,28,29,30,31]). Hence, it has become increasingly possible to fill in the knowledge gap between molecular evolution and field adaptation.

Within this context, we report phylogenetic studies, biochemical characterization, and three-dimensional structures of the α-amylase from *D. melanogaster*, which is an iconic model system in biology. Moreover, we characterized the inhibitory effect of potential proteinaceous inhibitors.

## 2. Results

*Drosophila melanogaster* α-amylase (Uniprot P08144), DMA, which has been classified into subfamily 15 of glycoside hydrolase family 13 (GH13_15) in the CAZy database, was studied from a biochemical and structural biology point of view in order to gain insights into the relationships between the structure, function and activity of this enzyme.

### 2.1. Three-Dimensional Structure of DMA

Crystals of DMA belong to the monoclinic space group *P*2_1_, and two molecules of DMA are present in the asymmetric unit. Structure solution was performed by molecular replacement, using the known three-dimensional structure of TMA [31] with which DMA presents 63% sequence identity as a search model. The crystal structure of native DMA was determined to 2.5 Å resolution and consists of a polypeptide chain of 476 amino acid residues, two metal ions (in principle calcium, at least that bound in the conserved structural calcium binding site) substituted by strontium in the structure due to crystallization conditions), one chloride ion and a number of water molecules (see Table 1).

**Figure 2:**
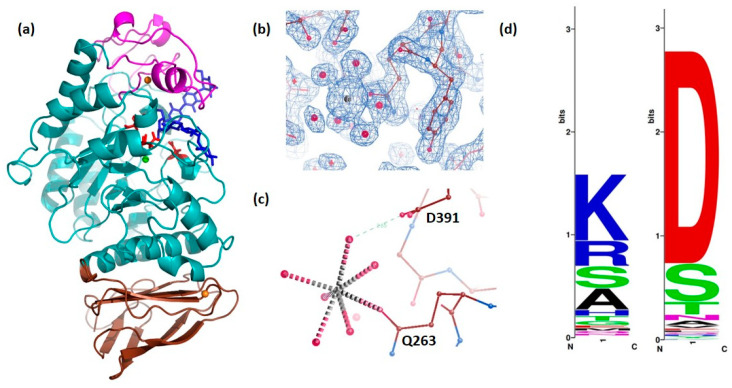
Overall structure of DMA (**a**) in a complex with the heptasaccharide product (molecule A): Domain A in cyan, domain B in pink, domain C in brown, orange spheres represent strontium ions (having replaced calcium at least for that bound between domains A and B), the green sphere presents a chloride ion and the heptasaccharide is shown in blue sticks. The three invariant essential catalytic residues in the active site, Asp186, Glu223 and Asp288 are highlighted as red sticks. Drawing made with PyMol (Schrödinger, LLC). (**b**) 2*F_o_-F_c_* density (contoured at 1σ) of the extra and nearly fully solvated strontium ion (putatively replacing a calcium ion) (**c**) interactions of the extra Sr^2+^—ion in the additional cation binding site (**d**) Weblogo diagram (http://weblogo.berkeley.edu/logo.cgi (accessed on 25 October 2022) [45]) indicating that Asp391 (to the right) is conserved in all Drosophila except *D. pseudoobscura*, and present in some other animal α-amylases, whereas Gln263 (to the left) is weakly conserved.

The overall dimensions of a DMA monomer are approximately 77 Å × 44 Å × 43 Å. As reported for other α-amylases from mammals [16,22,33], insect [34], plants [35,36], fungi [37,38], archaea [39] and bacterial α-amylases [25,40,41,42,43,44], the enzyme consists of three distinct domains, A (residues 1 to 97 and 160 to 379), B (residues 98 to 159) and C (residues 380 to 476). DMA contains eight cysteines which all are involved in intra-domain disulfide bridges, and which include Cys46–Cys102, Cys153–Cys167, Cys376–Cys382 and Cys448–Cys460. The crystal structure is shown in Figure 2a. Domain A, which hosts the active site of the enzyme, displays a (β/α)_8_-barrel fold. Domain B is characterized by its high variability in α-amylases depending on the origin, and as in other reported structures it is flanked by β3 and α3, and participates in binding a conserved calcium ion (here substituted with strontium due to crystallization conditions) at the interface with domain A. This metal ion contributes to the enzyme’s structural integrity. Domain C in the C-terminal part of the protein is composed of an eight-stranded anti-parallel β-sheet. Apart from the above mentioned conserved structural calcium ion, a chloride ion is located in the vicinity of the active site (Figure 2a) and a second non-conserved and highly solvated metal ion was identified in the electron density (see Figure 2b,c).

By analogy with other GH13 enzymes, the active site residues could be identified. In DMA, the nucleophile is Asp186, the general acid/base catalyst Glu223 and the transition state stabilizer is Asp288 (Figure 2a). In DMA the conserved calcium ion is coordinated by three water molecules, and residues from domain A (Asn98 OD1, Arg147 O, Asp156 OD1 and Asp156 OD2) and from domain B His190 O. Arg147 belongs to a well conserved motif and is present in more than 300 known animal Amy sequences, but not in *Caenorhabditis elegans* (Lys), nor in the paralog Amyrel present in true flies (Muscomorpha), which has a glutamine instead [46] (see Figure 1). His190 is conserved in all α-amylases except those from plants, where it has been substituted by glycine. As previously reported [17], the conservation of this residue is related to its interaction with the sugar moiety of the oligosaccharide lodged in subsite +1 of the active site (see Supplementary Figure S1).

As opposed to the structural calcium ion which is highly conserved in α-amylases whatever their origin, crystal structure analyses have shown that the chloride ion is present mainly in mammalian α-amylases (classified into subfamily GH13_24) and in *Pseudoalteromonas haloplanktis* α-amylase, AHA—GH13_32 [28,47,48], and the insect α-amylase TMA GH13_15 [34]. In the DMA three-dimensional structure a chloride anion is present and is coordinated by Arg184 and Asn286 and Arg325. A second shell of amino acid residues performing indirect interactions with the chloride anion, through hydrophobic forces, includes mainly the well conserved Leu281 and the invariant Phe246, but also the catalytic general acid–base Glu223. In the crystal structure, the distance between the chloride activator ion and the water molecule which is suggested to be involved in the hydrolytic reaction is 4.7 Å as is also seen in other chloride dependent α-amylase crystal structures such as, e.g., in AHA [27].

The non-conserved metal ion which has been identified in the electron density as described above is highly solvated, being ligated to six water molecules, and performs only one direct coordination bond to the enzyme Gln263 which is weakly conserved, even within Drosphila (Figure 2c,d). Apart from this direct coordination, the only contact this ion has with DMA is via a water molecule to Asp391 (Figure 2c), a residue which is conserved in all Drosophila except *D. pseudoobscura* (Figure 2d).

### 2.2. Three-Dimensional Structure of DMA in Complex with Acarbose

**Figure 3:**
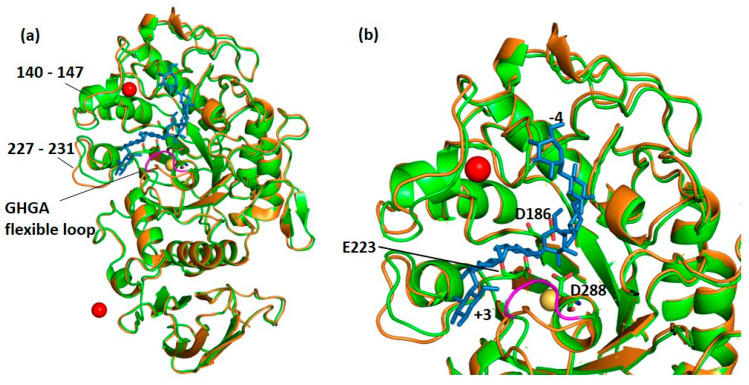
(**a**) Superposition of the native structure of DMA in orange onto that of the complex (green) with acarbose (blue sticks). It can be observed that the flexible loop (colored in magenta) is displaced upon binding of the inhibitor. (**b**) Close-up on the active site of DMA with the transglycosylation product (in blue sticks) occupying sub-sites −4 to +3, the three essential catalytic amino acids Asp186 (nucleophile), Glu223 (general acid/base) and Asp288 (transition-state stabilizer) presented as sticks), the chloride ion (yellow sphere) and the strontium ion which has replaced the so-called structural calcium ion in red.

The three-dimensional structure of DMA in complex with the well-known α-glucosidase inhibitor acarbose was solved to 2.0 Å resolution. Several studies have reported transglycosylation reactions taking place within α-amylase crystals, in particular when using the pseudo-tetrasaccharide acarbose as ligand [23,27,43,49,50,51,52,53,54,55]. For the DMA-acarbose complex, careful inspection of the electron density maps in the active site region revealed a well-defined heptasaccharide occupying subsites −4 to +3 resulting from a transglycosylation reaction as seen for the psychrophilic AHA [27] and the human salivary α-amylase, HSA [56]. The transglycosylation product and interacting amino acid residues are shown in Supplementary Figure S1. Upon binding of the heptasaccharide product (Figure 3a) the flexible loop, which proceeds the transition state stabilizer Asp288, undergoes movement as seen in Figure 3b.

### 2.3. Biochemical Properties

**Figure 4:**
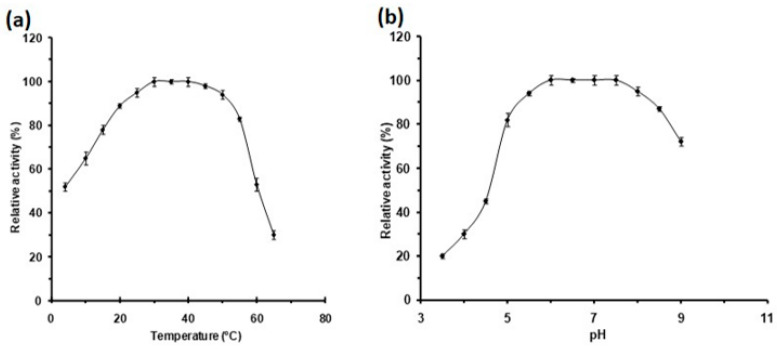
Activity profiles of DMA as a function (**a**) of temperature and (**b**) of pH. Activities at optimal pH and temperature (165 ± 3 U/mg) were defined as 100%. Error bars represent the standard deviation from three separate experiments.

The study of the DMA activity as a function of temperature indicated that the apparent temperature optimum covers a broad range from 30 to 45 °C. Moreover, this protein remained highly active at low temperatures, close to the ones encountered by the flies, since *D. melanogaster* remains active and feeds at about 12 °C (J.R. David, personal communication). Indeed, nearly 50, 65 and 80% of its activity was preserved at 4, 10 and 15 °C, respectively (Figure 4a). At 50 and 55 °C the protein retained 90 and 85% of its activity, respectively, whereas, at higher temperatures the relative activity values decreased. Hence, this protein maintains more than 75% of its maximal activity over a wide range of temperatures from 15 to 55 °C. The majority of α-amylases from *Drosophila* are optimally active at temperatures around 35 °C, far above the optimal life thermal range. Remarkably, α-amylases encoded by *D. pseudoobscura* alleles *Amy 1 and Amy 0.84* are optimally active at lower temperatures evaluated at 20–25 °C [57], whereas that of *D. virilis* is fairly active at 45 °C [58]. Our results revealed the wide apparent activity temperature range of DMA, which encompasses the activity range of all previously characterized *Drosophila* α-amylases. When studying the effect of pH on the DMA activity we observed that this enzyme was highly active with an apparent optimal pH 6.0–7.5 (Figure 4b). Furthermore, it should be noticed that the enzyme retained more than 80% of its activity at pH values from 5.0 to 8.5. Interestingly, this protein preserves more than 40% activity at pH 4.5, while a number of the reported α-amylases have optimal pH values of 7.0–8.0, or at alkaline pHs as, e.g., those from butterflies.

**Figure 5:**
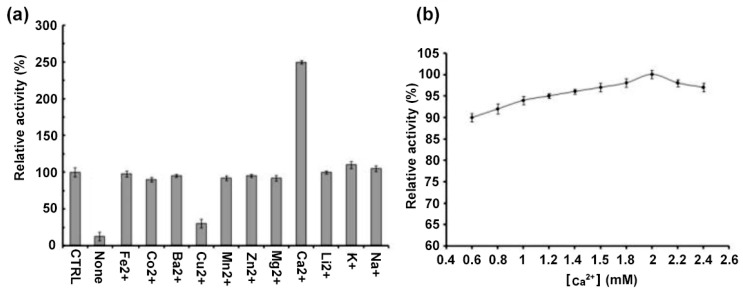
Activity profiles of DMA as a function of (**a**) the nature of divalent metal ions, (**b**) the concentrations of Ca^2+^. “CTRL” (control): purified DMA in its stock solution; none: enzyme after EDTA treatment to which no metal ions have been added. Activities at the intrinsic metal (calcium) ion concentration, “CTRL” (165 ± 3 U/mg) was defined as 100%. Error bars represent the standard deviation from three separate experiments.

To explore the effect of metal ions on the DMA activity, measurements were performed before and after EDTA treatment, resulting in a 90% decrease in the activity when treated with EDTA (Figure 5a) confirming that DMA requires metal ions for optimal activity. Amongst the tested metal ions only Ca^2+^ restored the enzyme activity which exceeded that observed for the metal depleted enzyme 2.5-fold (Figure 5a). Analysis of the role of the Ca^2+^ concentration revealed that for its maximal activity DMA needs only 2 mM Ca^2+^ (Figure 5b). Comparatively to previously reported α-amylases from other *Drosophila* species, the enzyme studied herein showed only a restricted requirement of metal ions for optimal activity, as opposed to those of *D. repleta* and *D. virilis* which require Ca^2+^, Na^+^ and K^+^ for their maximal activity [58]. Whereas Ca^2+^ restored the enzymes activity, Cu^2+^ has an inhibiting effect in accordance with earlier reported studies [59].

Enzyme kinetic studies showed that DMA has an affinity towards starch evaluated at 0.68 g L^−1^ (Table 2, Supplementary Figure S2). Analysis of the enzyme affinity for different substrates indicated that *K*_m_ values increased with decreasing sugar chain length, indicating that DMA affinity was higher towards longer sugar molecule chains. These data were confirmed by the catalytic efficiency measurements which demonstrate that DMA is more efficient on longer sugar chains.

### 2.4. Thermal and pH Stability

Study of the enzyme thermostability demonstrated that this protein was highly thermostable at temperatures up to 30 °C (Table 3). At 35, 40 and 45 °C the enzyme half-lives were decreased to 40, 38 and 29 h, whereas at higher temperatures including 50, 55 and 60 °C the enzyme half-lives dropped to 6, 4 and 0.9 h, respectively (Table 3). These results revealed that DMA was remarkably stable at temperatures up to 40 °C, which suggests this organism is adapted to a broad range of temperatures. Analysis of the enzyme stability at different pH demonstrates that this enzyme has high half-life values over a wide range of pH from 4.5 to 8.5 (Table 3). The half-life drops quickly below pH 4.5 and more slightly at pH 9. Such a large thermal (and pH) range of activity and stability can be advantageous in temperate regions, where flies experience important seasonal temperature variations.

### 2.5. Role of the Flexible Loop

Considering the frequent loss of the aforementioned flexible loop in the course of evolution, we assessed the role of the presence or lack of the GHGA motif in this loop (amino acids 292–295 in DMA, Figure 1 and Figure 3) on the activity and on the sensitivity to proteinaceous inhibitors.

The activity of native DMA was measured and compared with that of a deletion mutant (hereafter DMA_ΔGHGA) that lacked this motif. In an earlier study, Ramasubbu and colleagues [56] reported that in human salivary α-amylase (HSA), the histidine corresponding to residue His293 in DMA within this motif was important for catalytic activity. Our results are in agreement with this observation in that the deletion mutant is ten-fold less active on starch than the native enzyme (Supplementary Figure S3), but displays similar affinity (the *K*_m_ values were 1.86 ± 0.14 g L^−1^ (DMA) and 1.78 ± 0.14 g L^−1^ (DMA_ΔGHGA), respectively. In order to determine whether there was consensus for this within other animal α-amylases from the GH13_24 subfamily and holding this flexible loop, we produced a similar deletion mutant in porcine pancreatic α-amylase (PPA) and performed the same studies. Our results indicated that PPA_ΔGHGA displays even lower activity than what was observed for DMA_ΔGHGA (Supplementary Figure S3).

**Figure 6:**
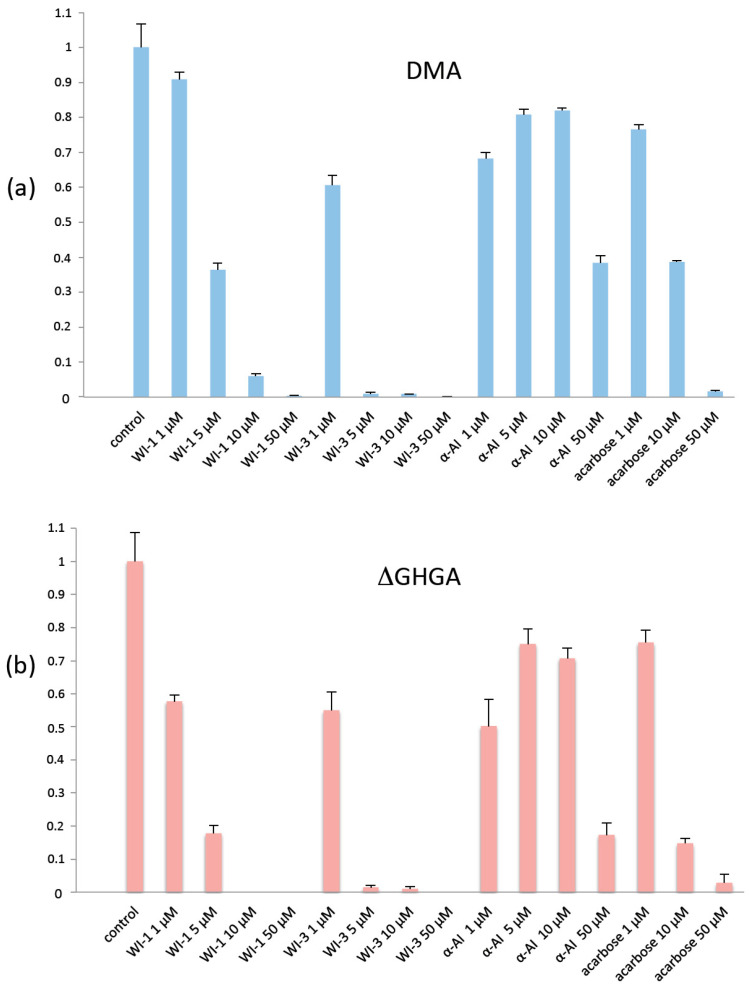
Residual activity of (**a**) native DMA after treatment with proteinaceous inhibitors and acarbose, as compared to (**b**) its deletion mutant DMA_ΔGHGA, which lacks the GHGA motif of the flexible loop. Control: no inhibitor, error bars represent the standard error of the mean from triplicate measurements. WI, wheat inhibitor and α-AI, *Phaseolus vulgaris* α-amylase inhibitor.

To test the effect of the presence or absence of the flexible loop on the sensitivity of DMA to inhibitors, we measured the inhibitory capacity of three known proteinaceous plant α-amylase inhibitors: α-AI from seeds of the common bean *Phaseolus vulgaris*, WI-1 (tetrameric form) and WI-3 (monomeric form) from *Triticum aestivum* seeds, on native DMA and on DMA_ΔGHGA. The activities in the presence of the inhibitor acarbose used for the previously mentioned complex, was measured and the results from all assays at 25 °C at physiological pH 7.5, following an earlier described method [60] are given in Figure 6. Wheat inhibitors were both efficient on DMA, especially WI-3, with residual activity of 6% for WI-1 and 0.8% for WI-3 at 10 μM. In contrast, for α-AI from *Phaseolus vulgaris*, even at the highest concentration (50 μM), the residual activity remained significant (38%). In earlier studies, Pytelkova and co-workers [61] have shown that the *Phaseolus vulgaris* α-AI was inefficient on the three α-amylases of the moth *Ephestia kuhniella*. Drosophilids and moths do not feed on seeds, so this result could be expected since there has been no selective pressure on the bean inhibitor to become efficient against those species. Interestingly, the deletion mutant DMA_ΔGHGA showed a higher sensitivity to the tested inhibitors, especially for WI-1 and acarbose (from 10 µM of this latter) and at higher concentrations of *P. vulgaris* α-AI as seen in Figure 6.

### 2.6. Comparative Studies with Other α-Amylases

DMA is the second enzyme of the GH13_15 subfamily for which a three-dimensional structure has been determined. When comparing the crystal structures with that of the previously solved TMA it can be seen that the overall fold is highly similar with an RMSD of 0.648 Å based on 432 Cα atoms, and that the differences in the fold are found in the loop regions as indicated in Supplementary Figure S4. The surface area of DMA is 47,166 Å^2^, compared to 46,195 Å^2^ for TMA, and the solvent accessible surface areas are 18,118 Å^2^ and 16,685 Å^2^, respectively. Whereas TMA, as earlier mentioned, displays the structural calcium ion and the chloride ion, the newly revealed metal ion situated between domains A and C in DMA is lacking in TMA (Supplementary Figure S5). Interestingly, when examining other α-amylases which need to cope with temperature adaptation, no metal ion binding sites are observed between domains A and C in the psychrophilic *Pseudoalteromonas haloplanktis*, GH13_32) α-amylase, AHA, whereas in both the thermostable *B. licheniformis* (GH13_5)—and the hyperthermophilic *P. woesei* α-amylases (GH13_7)—a calcium ion and a zinc ion, respectively, are binding to sites in the vicinity of the site hosting the new putative calcium observed in DMA.

Metal ions are not the only features nature has found to stabilize proteins. When studying the number of hydrogen bonding networks as a function of the number of residues in five α-amylases known to work at different temperatures, the psychrophilic enzyme clearly has the lowest number. These networks are known to stabilize the overall structure of proteins, and interestingly, DMA contains an approximately 30% higher hydrogen bonding network than the psychrophilic enzyme, but 13 and 25% lower than the hyperthermophilic and thermostable enzymes, respectively. Even more intriguingly, no hydrogen bonding network is observed (as calculated with ProteinTools [62]) in/to domain C for AHA, whereas for the other enzymes the network spans the entire enzyme structure being most flared out in the thermostable and hyperthermostable α-amylases. The insect enzymes DMA and TMA fall somewhat in between this pattern (Supplementary Figure S6). When looking in detail at the hydrogen bonding network pattern in domain C, it is more spreadout in TMA than in DMA, which instead displays a metal binding site (newly observed putative calcium ion as discussed above) in the region lacking hydrogen bonding networks as compared to TMA.

Another stabilizing feature of protein structures are salt-bridges, and the number of salt-bridges present at the surface tends to increase with thermostability. In a comparative study of the five above mentioned α–amylases, the number of salt-bridges (calculated with ProteinTools [62]) of DMA is comparative to that of the cold-adapted AHA, and lower than that of the other enzymes. While the thermostable and hyperthermophile display salt-bridges covering the entire structure of the enzymes and the enzyme surfaces (Supplementary Figures S7 and S8), those of the psychrophilic AHA, of TMA and DMA seem more localized. Additionally, it should be highlighted that in DMA no salt-bridges are observed between domains A and C. Finally, the number and distribution of hydrophobic clusters exposed to the solvent were analyzed, and no significant differences were observed (Supplementary Figure S9).

DMA displays the previously mentioned flexible loop, whereas TMA does not (Figure 1). When superimposing TMA onto DMA this is readily visible in the structure as seen in Supplementary Figure S4.

### 2.7. Docking Studies of DMA and Insect Inhibitors

**Figure 7:**
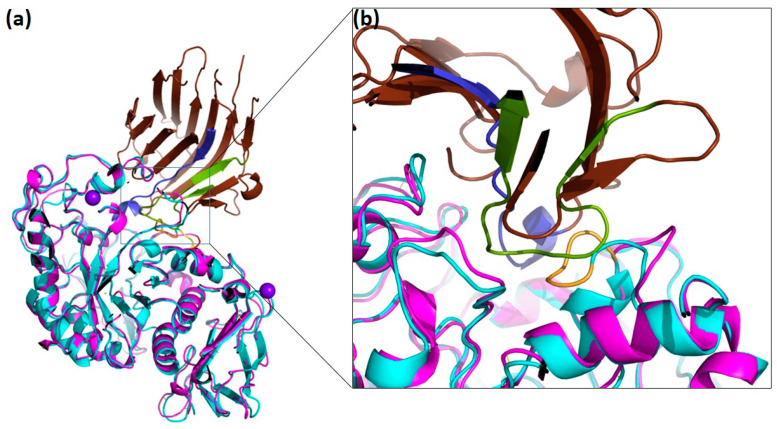
Docking of the three-dimensional structure of DMA with *P. vulgaris* α-AI. (**a**) Native DMA (cyan) with its metal ions in purple has been superposed onto TMA (pink) from the TMA/α-AI complex (PDB code 1VIW, [63]). The inhibitor is colored in brown, with hairpin loops 1 and 2 colored in green and blue, respectively. (**b**) Close-up of the interaction interface and α-AI with the flexible loop (yellow) in DMA.

Following the inhibition studies using the three plant α-amylase inhibitors α-AI, WI-1 and WI-3 on DMA, we performed docking studies in which the three-dimensional structure of DMA was superimposed onto TMA of the TMA/α-AI complex (Figure 7, Supplementary Figures S10 and S11). As depicted in Figure 7, it can be observed that the flexible loop corresponding to residues Gly292—Ala295 which has been deleted in the deletion mutant DMA_ΔGHGA collides with a part of hairpin loop 1 which performs interactions with TMA in the TMA/α-AI complex [63], and comes too close to Ser189 of α-AI. As concerns WI-1 and WI-3, no experimental three-dimensional structures have been determined to date.

### 2.8. Phylogenetic Studies

In contrast to the previously described *T. molitor* insect α-amylase (TMA), and as mentioned above, the crystal structure of DMA determined within these studies revealed a novel metal binding site located at the interface between domains A and C (Figure 2a–c). This newly observed putative Ca^2+^ ion is coordinated by Gln263 (domain A) and six water molecules, of which one is bound to Asp391 (domain C). We have analyzed the phylogenetic conservation of these two amino acids across animals. Asp391 appears to be well conserved in animals, whereas the occurrence of glutamine at position 263 is rare (Figure 2d). If focusing on insects, in a sample of 129 insect sequences, Asp391 accounts for 72.9% (Supplementary Figure S12). Interestingly, TMA has a serine residue at this position. In contrast, similar to non-insect animals, Gln263 is weakly conserved, and the occurrence of a glutamine is mainly restricted to the close relatives of *D. melanogaster*, an arginine being more likely ancestral in drosophilids.

### 2.9. Enzyme Variability in Drosophila melanogaster

The *melanogaster* subgroup of Drosophila species is a suitable model for studying the molecular adaptation of α-amylase, as it includes generalist and specialist species, endemic and invasive species. The biochemical properties of some of these were roughly compared using crude extract by Shibata and Yamazaki [64] and more recently with purified recombinant enzymes by Commin and colleagues [65]. Indeed, sequence comparative studies of these species may enable the exploration of potential adaptive features of amino acid substitutions.

At the intraspecific level, in *D. melanogaster*, the selectionist hypothesis posited that the enzyme variants were under natural selection, and it was therefore assumed that each variant would bear particular fine adaptations. However, pioneer biochemical studies, e.g., those communicated by Doane [19], were not conclusive because each genotype contained a mixture of different electrophoretic variants, owing to the gene being duplicated in *D. melanogaster*. Therefore, we sampled 72 sequences, including DMA (BAB32511); 27 amino acid positions were variable, about one-third of which affected the global charge (Supplementary Figure S13). This highlights that the classical electrophoretic techniques have disclosed only a part of the diversity and thus the adaptive ability of this enzyme [66]. Araki and colleagues [67] found positive selection at seven amino acid positions in Amy sequences of *D. melanogaster*, compared to its close relative *D. simulans*. However, our reconstruction of the ancestral α-amylase sequence of Drosophila showed that most of the substitutions identified in this work [67] in *D. melanogaster* were likely reversions to the ancestral state (Supplementary Table S1).

## 3. Discussion

As other GH13 family members, DMA is a retaining glycoside hydrolase which operates by a double displacement mechanism with the formation and breakdown of a covalent glycosyl-enzyme intermediate via an oxocarbenium ion-like intermediate, a mechanism which is relatively well established (see, e.g., [68,69]). As observed for other α-amylases, this insect α-amylase performs transglycosylation reactions when in the presence of the anti-diabetes pseudo-tetrasaccharide drug, acarbose, under water poor conditions. Hereby, the pseudo-tetrasaccharide which is synthesized by *Actinoplanes* sp. and which is composed of an acarviosin linked to maltose, is transformed to a heptasaccharide consisting of an acarviosin-glucose linked to an acarbose (Figure 2a and Figure 3, Supplementary Figure S1).

All existing three-dimensional structures of α-amylases corroborate that nearly all α-amylases display at least one tightly bound Ca^2+^ ion which is involved in catalysis as well as in the structural architecture of the protein [48]. In addition to this structural calcium ion, a second metal ion, which is located between the catalytic domain A and domain C, was revealed in the DMA crystal structures described herein. It is interesting to notice that the only direct coordination of this putative calcium ion to the protein is via Gln263, an amino acid residue which is only conserved in the subgroup of *D. melanogaster* and which is not present in other animal α-amylases. Besides this, the newly discovered metal ion is coordinated to water molecules. As a curiosity, an earlier study of the crystal structure of the protein–protein complex between isozyme 2 barley α-amylase (AMY2) and the endogenous protein inhibitor BASI, identified an interfacial fully solvated calcium ion [70] which, however, was not observed in the native crystal structure of AMY2. The role of the newly revealed metal ion in DMA remains unknown, but one may speculate that given the sole conservation of Gln263 within the *D. melanogaster* subgroup, it may be an adaptive response to the relatively large temperature variations that the fly has to cope with. Indeed, the presence of divalent ions (Ca^2+^ amongst others) between domains A and C (and in this region) has been observed in earlier studies of the thermostable *Bacillus licheniformis* α-amylase [71], and a zinc ion was bound to domain C of the hyperthermophilic *Pyrococcus woesei* α-amylase [72].

Some α-amylases have been reported to be allosterically activated by chloride ions [47], including those from animals, but also from several gram-negative micro-organisms such as, e.g., *Pseudoalteromonas haloplanktis* [73]. In all known crystal structures of chloride-dependent α-amylases, this anion was located in the vicinity of the active site, and this is also the case in the crystal structure of DMA. The location of this chloride ion and its role in the enzymatic activity, clearly shows that this anion binding is designed to adjust the properties of active site residues to promote catalytic activity [29,74]. Maurus and colleagues [74] reported the crucial role of Asn298 in human pancreatic α-amylase (Asn286 in DMA) in association with the chloride ion on modulation of catalysis, the latter influencing the orientation of the catalytic proton donor Glu233. Their studies indicated that when the chloride ligand Asn298 is mutated to serine, the latter interacts with the transition state stabilizer, and thereby alters the conformation of the flexible loop typical of chloride-dependent mammalian α-amylases, and studied within the current work. Structural studies indicated the high conservation of two of the chloride ligands, Arg183 and Asn286 in DMA, in all chloride-independent α-amylases (see, e.g., [28]). In contrast, the third chloride binding ligand (Arg325 in DMA as in mammalian α-amylases) is substituted by a lysine in *P. haloplanktis* and in several beetle α-amylases (e.g., *Anthonomus grandis*, *Ips typographus*), and by a glutamine in Lepidoptera, in the cicada (Hemiptera) *Tamasa doddi* and in the α-amylase paralog Amyrel of some dipteran species. It has been suggested that for chloride-independent α-amylases, the lack of this ion is compensated by a hydrophobic core which modifies the substrate interactions and the position of Asn298 [74]. The role of chloride ions in DMA seems to be equivalent to what is proposed for mammalian α-amylases which display identical anion coordinating amino acid residues. One may speculate that it could have a regulating role in promoting activity at lower temperatures, and/or that it could be involved in the up or downregulation of the various isozyme activities depending on the environment and thereby the availability of substrate.

Since α-amylases play an essential role in insect survival, they constitute appealing protein targets to fight insect pests. Our studies confirmed that native DMA is inhibited by the wheat inhibitors WI-3 and WI-1 but conserved 38% of its residual activity when in presence of the *Phaseolus vulgaris* inhibitor α-AI. Intriguingly, shortening of the flexible loop in the deletion mutant DMA_ΔGHGA resulted in clearly decreased activity in the presence of WI-1, whereas it had no effect on WI-3. In the presence of α-AI, the activity was affected particularly at higher inhibitor concentrations. These observations indicating higher sensitivity of the shortened-loop variant to seed inhibitors is in agreement with the fact that those inhibitors were likely shaped by evolution against coleopteran α-amylases which are the natural targets, and which have a shortened flexible loop, here mimicked by our deletion mutant. It should be noted here that, apart from the beetles, the flexible loop was deleted independently several times across insect evolution, and hence there is no correlation between this feature and insect taxonomy. Supplementary Figures S14 and S15 illustrate the occurrences of the losses in insects and in Diptera, respectively. A correlation with the ecology of the species remains a valuable hypothesis, and the contrasted responses to inhibitors between the wild-type DMA and the DMA_ΔGHGA mutant constitute interesting clues hinting toward a selective pressure from food. These results suggest different interaction modes of DMA with the respective wheat inhibitors (WI-3 and WI-1), and that shortening of the flexible loop presumably results in a larger interface between DMA and WI-1. As concerns the low inhibition effect of α-AI on DMA, this could partly be due to residues Ala298 and Asp299 of the DMA flexible loop not interacting with hairpin loop 1 (residues 29–46) as opposed to TMA. Indeed, if superimposing the DMA structure onto TMA in the complex between TMA/α-AI [63], these two residues from the flexible loop occupy the positions of Ser294 and Asn295, respectively, in the TMA three-dimensional structure. Moreover, in DMA, the deleted residues of the flexible loop, Gly292 to Ala295, together with Gly296 and Gly 297 collide with a part of hairpin loop 1. One may speculate that the reduced activity of the deletion mutant in the presence of α-AI when compared to that of the native enzyme could be a result of an increased interaction surface due to less steric hindrance when deleting the four residues in the deletion mutant DMA_ΔGHGA. Finally, it is interesting to notice that the position of the newly identified metal ion in the DMA structure is at the same site where α-AI binds, if assuming that the inhibitor binds in a similar manner to DMA, as seen for TMA. This raises the question of whether this putative calcium plays a role in the contact to, e.g., the other proteinaceous inhibitors as was observed in the AMY2/BASI complex [70], and should be the subject of future investigations.

Our enzyme activity studies disclosed that DMA displays a wide optimum pH (6–7.5), and that more than 80% of the relative activity was conserved between pH 5 and 9. Interestingly, the Drosophila larval midgut is defined by five regions of pH, including the acidic mid-midgut which is the only part in which DMA is not expressed [75,76]. The other parts of the midgut (anterior- and posterior midgut) exhibit pH values from pH 6 to 9.5. The insect enzyme was moreover, remarkably stable across a broad temperature range and up to 40 °C. Both activity studies are thus fully in agreement with the physiological conditions of Drosophila and the need for adaptation to various environments. Interestingly, upon comparison of the hydrogen bonding network pattern of DMA, with the four other α-amylases which are adapted to different temperatures, and which were discussed earlier, the spread/distribution of hydrogen bonds within the respective three-dimensional structures seems to increase with increasing temperature. DMA which was found to be active over a large temperature range, displays a hydrogen bonding network distribution pattern being situated in between those observed for the psychrophilic enzyme, and those observed for thermostable and hyperthermophile enzymes, and which is supportive of the need for adaptation of the enzyme to a broad temperature range.

Regarding thermal adaptation, Prigent et al. [77] observed a correlation between the global charge of drosophila amylases and the climate in which the flies live (temperate vs. tropical). They collected electrophoresis data for more than one hundred Drosophila species. The electric charge was more negative in tropical areas than in temperate regions. The authors hypothesized that “an increase in negative amino acids is likely to improve amylase stability in tropical areas”. In *D. melanogaster*, six major electromorphs (i.e., electric charge variants) were described [19] with pI’s ranging from 5.40 to 6.23. Since all were of tropical African origin [78], these diversely charged enzymes should experience similarly warm temperatures. Thus, it is difficult to assume what was earlier suggested by Prigent et al. [58], that the occurrence of diversely charged alleles resulted from the selective action of local temperature on charged amino acids towards better thermal stability. The reason for which the most frequent isozyme, Amy1, the electrophoretic class to which DMA belongs, has spread worldwide at a high frequency, might be due to a founding effect, but also to selection of regulatory elements linked to Amy1, acting on amylase expression, related to, e.g., glucose content in the food available [79]. In any case, the large thermal range of DMA activity evidenced from our current studies is advantageous in temperate regions, where flies experience important seasonal temperature variations.

Positive selection was found in Amy sequences of *D. melanogaster*, compared to its close relative *D. simulans* [67]. In that study, the authors concluded that most amino acid substitutions took place in the *D. melanogaster* lineage soon after speciation and were adaptive, leading to a “much higher activity due to one or more of these substitutions”. However, this higher activity [64] was relative to the total protein content, without knowledge of α-amylase content percentage of the latter.

## 4. Materials and Methods

### 4.1. Bacterial Strains, Media, Plasmids and Growth Conditions

*Escherichia coli* BL21 was used as the mutant host strain and culture of *E. coli* strains, was performed in Luria Bertani (LB) medium [80] at 37 °C and supplemented, when necessary, with ampicillin (100 µg/mL) and IPTG (160 µg/mL). The *amy-p* gene (coding for DMA) was cloned in the pGEM-T Easy vector (Promega, Madison, WI, USA). pET22b was purchased from Novagen and used as recommended by the manufacturers, and the pET-DMA plasmid harbors the *amy-p* gene, encoding *Drosophila melanogaster* α-amylase, under the control of the T7 promoter.

### 4.2. Over-Expression and Purification of Drosophila melanogaster α-Amylase

The pGEM-T-DMA plasmid was digested with NdeI and HindIII restriction enzymes. The resulting DNA fragment, harboring the *amy-p* encoding gene, was sub-cloned in the pET22b vector, giving the pET-DMA plasmid. In this construction *amy-p* was placed downstream of the T7 promoter.

For purification, a 1 L culture of the recombinant strain BL21/pET-DMA was produced under the abovementioned conditions. After centrifugation, proteins in the supernatant were concentrated by ammonium sulphate precipitation at 80% saturation. After 1 h of precipitation, the suspension was centrifuged at 13,000× *g* for 50 min and the pellet re-suspended in 70 mL of buffer A (50 mM Tris, pH 7.5 containing 1 mM CaCl_2_) and dialyzed against 2 × 2 L of buffer B (20 mM HEPES, pH 7.5 containing 20 mM NaCl) for 24 h.

Cooled, pure ethanol (final concentration of 40%) was added drop wise to the solution which was stored on ice with constant stirring for 40 min. Then, the suspension was centrifuged at 28,000× *g* for 10 min to eliminate precipitate which mainly contained nucleic acids. Amylolytic activity in the supernatant was determined and glycogen was added to a final glycogen to enzyme ratio (*w*/*w*) of 4:1, followed by centrifugation of the mixture as described above to precipitate the enzyme–glycogen complex. Residual activity in the supernatant was monitored and when necessary, the precipitation step was repeated. Finally, the pooled pellets were re-suspended in 100 mL of buffer A and dialyzed against 2 × 2 L of the same buffer for 24 h, followed by three chromatography steps. First, the protein solution was loaded onto a Q-Sepharose anion exchange column and eluted with a linear NaCl gradient (0–0.4 M) in the same buffer. Protein fractions displaying amylolytic activity were concentrated to 10 mL by ultrafiltration on a Millipore polyethersulfone membrane with a 10 kDa cut-off in an Amicon Ultrafiltration cell under a nitrogen pressure of 3 bars. The concentrated sample was loaded onto a Sephadex G100 gel filtration matrix and eluted with 500 mL of buffer A. The affinity of the α-amylase for this matrix was used to remove protein contaminants of similar size. Fractions of interest were concentrated to 10 mL by ultrafiltration as described above. In a final chromatographic step using Ultrogel AcA54, non-proteinaceous contaminants such as salts and nucleic acids were eliminated. This polishing step also allowed changing from Tris buffer to MOPS buffer (10 mM MOPS, 10 mM NaCl, 0.5 mM CaCl_2_, pH 7.5), for crystallization experiments and enzyme kinetic studies.

Native and mutant α-amylases used for the measurement of residual activity in the presence of inhibitors were produced in *Pichia pastoris* strains GS115 or KM71 (Invitrogen Pichia multi-copy expression kit) and glycogen-purified according to Commin et al. [65], an earlier study, which indicated that there was no significant difference between the enzymes produced either in *E. coli* or *P. pastoris*, although the latter may be glycosylated. The GHGA motif deletion mutant was obtained by inverse PCR using the Q5-directed mutagenesis kit (New England Biolabs, Ipswitch, MA, USA).

### 4.3. Crystallization

Screening of DMA crystallization conditions were carried out using the hanging-drop vapor diffusion method, and commercially available crystallization screening kits. Crystallization using a solution of 20% (*v*/*v*) PEG monomethyl ether 550, 0.1 M sodium chloride and 0.1 M bicine buffer (pH 9.0) resulted in a shower of small needle-shaped crystals at room temperature. The protein to mother liquor ratio was 1:1 in 2 µL drops (initial protein concentration was 22.8 mg/mL) equilibrated over 500 µL reservoir solution. These conditions were further optimized using the additive screen kits from Hampton Research (Hampton Research, Aliso Viejo, CA, USA). Crystals suitable for X-ray diffraction studies were obtained after approximately one week in 20% (*v*/*v*) PEG monomethyl ether 550, 0.1 M sodium chloride, 0.1 M bicine buffer (pH 9.0) and 10 mM strontium chloride, and a single crystal was used for collection of the native data. For the complex with acarbose, crystals used for data collection were soaked in the crystallization buffer supplemented with acarbose to a final concentration of 10 mM for 1 h.

### 4.4. Data Collection

Crystals were cryoprotected by striking the cryo-loop (Hampton Research, Aliso Viejo, CA, USA) in which the crystal was mounted in a 100% ethylene glycol solution prior to crystal mounting, followed by flash-cooling in liquid nitrogen. Diffraction data were collected at the European Synchrotron Radiation Facility (ESRF) and data collection and refinement statistics are given in Table 1. Crystals belong to the monoclinic space group *P*2_1_ and reflections were indexed, integrated, scaled and merged using programs from the XDS package [81].

### 4.5. Structure Determination and Refinement

Phases and experimental electron density maps for native DMA were calculated after a molecular replacement search using the crystal structure of *T. molitor* larvae α-amylase at 1.65 Å resolution [34] as a search model with the PHASER [82] program. The initial model was built using Phenix AutoBuild [83]. Hereafter, alternating cycles of model building, and refinement were performed employing Coot [84] and REFMAC [85]/Phenix [86].

Two independent monomers are present in the asymmetric unit. The molecular replacement search included data to 3.5 Å resolution; in the remaining refinements data were extended to 2.5 Å. For the enzyme complex the crystal structure was solved using difference Fourier methods and the native structure of DMA as a starting model. Refinements (REFMAC [85]/Phenix [86]) were alternated with visual examination of electron density maps and manual building using the graphics software COOT v0.9.6 [84,87]. To avoid over-refinement, *R* and *R*_free_—factors [32] were monitored; *R*_free_ was calculated from a test set of 5% of the reflections randomly selected from all data. Based on inspection of 2*F_o_-F_c_* and *F_o_-F_c_* maps (contoured at 1 and 3σ, respectively), strontium and chloride ions were inserted, and water molecules were added, respecting hydrogen-bonding distances and angles. A model of the ligand from the HIC-UP database [88] was manually inserted in the electron density maps. Model qualities were examined with MolProbity [89] and WHATCHECK [90]. The models show good stereochemistry and refinement statistics as summarized in Table 1.

### 4.6. Bioinformatics and Figure Rendering

Protein sequence alignments were displayed and manually adjusted when necessary, using the Geneious 11.0.5 software (Biomatters Ltd., Auckland, New Zealand) after alignment with MAFFT [91], implemented in the same software.

The phylogenetic trees were computed using the IQ-TREE web server [92] with default parameters and 1000 ultrafast bootstraps, and drawn using iTOL [93].

Sequence logos of the conservation of amino acid positions homologous to Gln263 and Asp391 in 296 and 294 animal sequences, respectively, were displayed using the WebLogo resource (http://weblogo.berkeley.edu/logo.cgi (accessed on 25 October 2022)) [45].

Identification of hydrophobic clusters, hydrogen bonding networks and salt bridges were performed using the ProteinTools web server [62].

Rendering of figures was achieved using the programs PyMol Version 0.99, Schrödinger, LLC) and COOT [84,87].

Ancestral states at some amino acid positions were reconstructed with ASR, implemented in the HyPhy package [94] at www.datamonkey.org, using an alignment of *Drosophila* α-amylase sequences and the Automatic Model Selection. The GTR model was then used, and ancestral states were inferred using three likelihood methods [95,96,97] for the node basal to the subgenus *Sophophora*, to which *D. melanogaster* belongs.

### 4.7. Activity Measurements

The activity of DMA was determined by measuring the amount of reducing sugars released after incubation with soluble starch (Sigma-Aldrich, St Louis, MO, USA) dialyzed twice to remove metal ions. A 50 µL volume of enzyme solution at ca. 15–20 ng/µL was added to 500 µL of 1% (*w*/*v*) starch diluted in 100 mM MES buffer (pH 6.0), and samples were adjusted to 1 mL with buffer followed by incubation for 2 min at 35 °C. The amount of released reducing sugars was determined by the dinitrosalicylic acid method [98]. One unit of α-amylase activity was defined as the amount of enzyme releasing reducing sugars equivalent to 1 µmol of glucose per min under the specified condition assays. Residual activity measurements were performed in the presence of potential proteinaceous and acarbose inhibitors in 20 mM HEPES buffer at pH 7.5, 20 mM NaCl, 1 mM CaCl_2_. First, the enzyme and the inhibitor were incubated (10 µL each) for 20 min at 25 °C, then the activity was measured at 25 °C. The enzyme concentration prior to mixing was 0.6 µM (32 ng/µL) and the inhibitor concentrations ranged from 1 to 50 µM. Proteinaceous inhibitors were α-AI from seeds of the common bean *Phaseolus vulgaris* (MP Biomedicals, Illkirch, France, ref. 153877), whereas WI-1 (tetrameric form) and WI-3 (monomeric form) were from *Triticum aestivum* seeds (Sigma-Aldrich A1520 and A3535, respectively). Acarbose was from Sigma (A8980).

#### 4.7.1. Temperature, pH and Stability Profiles

The effect of temperature on the activity was determined by incubating the purified enzyme at temperatures ranging from 4 to 65 °C, whereas the DMA pH profile was obtained by measuring the activity at various pH values from 3.0 to 9.0 (3.0 to 5.0 with sodium acetate buffer, 6.0 to 7.0 with MES buffer and 7.5 to 9.0 with bicine buffer). The enzyme stability as a function of temperature and pH was measured by incubating the enzyme at different temperatures and pH, withdrawing the samples at defined intervals, placing them on ice and measuring residual activity at 35 °C and pH 6.0.

#### 4.7.2. Effect of Metal Ions

Purification of DMA was performed as described in the enzyme purification section but without adding metal ions to the buffer, and dialyzed for 48 h against 100 mM MES buffer (pH 6.0) containing 10 mM EDTA at 4 °C (buffer renewed twice). The enzyme was pre-incubated for 20 min in the presence of 1 mM CoCl_2_, MnCl_2_, ZnCl_2_, MgCl_2_, BaCl_2_, FeCl_2_, CaCl_2_, LiCl_2_, KCl, NaCl, CuCl_2_, respectively, and activity assays were performed under standard conditions. Determination of the optimal metal ion concentrations was performed by incubating the enzyme samples with metal ions at concentrations from 0.6 to 2.4 mM, followed by monitoring of the activity.

#### 4.7.3. Determination of Kinetic Parameters

Assays were carried out using a 100 mM MES buffer (pH 6.0), 2 mM CaCl_2_ and 0.2 to 80 g/L substrate and incubation for 2 min at 35 °C. The reaction was stopped by adding DNS followed by heating to 100 °C and the amount of released sugar was determined as described above.

## 5. Conclusions

DMA is the second insect α-amylase (after TMA), and the second subfamily GH13_15 member for which the three-dimensional structure has been solved. Together with the other existing three-dimensional structures of α-amylases, we now have a sound basis that will allow future work in silico as well as on the bench, to assess the effect of sequence variations that may be encountered in nature. In future studies, mapping of primary structure differences in variants of *D. melanogaster* onto the DMA three-dimensional structure template may give insight into evolution and environmental adaptation. Taking into consideration the preponderant role of this enzyme on the survival and growth of larval insects, the crystal structure of this enzyme is also highly interesting for contributing to conceiving inhibitors for combating insect pests. Our results reported herein could serve as the starting point for this purpose.

## Figures and Tables

**Table 1:** Data collection and refinement statistics.

	Native DMA	DMA/Acarbose
PDB-ID **Data collection**	8OR6	8ORP
Beamline	ID23-2	ID14-1
Temperature (K)	100	100
Wavelength (Å)	0.8726	0.9334
Resolution range (Å)	44.8–2.5	30.0–2.0
Space group	*P*2_1_	*P*2_1_
Cell dimensions		
a, b, c (Å)	a = 80.4, b = 73.1, c = 99.3	a = 80.1, b = 72.5, c = 98.2
α, β, γ (°)	β = 98.3	β = 98.4
n° reflections	148,467	280,188
n° unique reflections	39,454	75,240
*R*_merge_ (%)	21.6	13.5
CC1/2	0.967 (0.641)	0.993 (0.778)
I/σ(I)	6.4 (3.0)	9.0 (2.3)
Multiplicity	3.8	3.7
Completeness (%)	99.2 (93.9)	99.8 (99.7)
n° molecules/asymm. unit	2	2
**Refinement**		
*R*/*R*_free_ ^1^ (%)	19.0/27.6	17.6/21.2
n° atoms		
protein	7312	7318
water	493	874
ligands	-	304 ^3^
n° ions per molecule		
strontium ^2^	2	2
chloride	1	1
Average B-factor (Å^2^)		
protein	18.7	17.6
water	19.7	27.8
RMSD		
Bond lengths (Å)	0.002	0.003
Bond angles (°)	0.47	0.64
Ramachandran		
Favored (%)	97.2	97.5
Allowed (%)	2.6	2.3
Outliers (%)	0.2	0.2

^1^ *R*_free_ [32] is the *R*_factor_ calculated from 5% of the data which have been randomly selected and excluded from refinement. ^2^ Putative calcium ions have been substituted by strontium ions in the structures due to the crystallization conditions. ^3^ Two molecules of acarbose and 38 molecules of ethylene glycol (this latter used for cryoprotection).

**Table 2:** Kinetic parameters for the hydrolysis of starch and maltooligosaccharides by DMA. The experiment has been performed in triplicate.

**Substrate**	***K*_m_** **(g L^−1^)**	***k*_cat_** **(s^−1^)**	***k*_cat/_*K*_m_** **(s^−1^/g L^−1^)**
starch	0.68 ± 0.06	571 ± 1	840 ± 17
**Substrate**	***K*_m_** **(µM)**	***k*_cat_** **(s^−1^)**	***k*_cat/_*K*_m_** **(µM^−1^/s^−1^)**
maltotriose	94 ± 5	194 ± 6	2.1 ± 0.1
maltotetraose	70 ± 1	223 ± 3	3.2 ± 0.9
maltopentaose	68 ± 8	278 ± 2	4.1 ± 0.7
maltohexaose	61 ± 6	370 ± 4	6.1 ± 0.6
maltoheptaose	52 ± 4	493 ± 1	9.5 ± 0.4

**Table 3:** Enzyme stability of DMA as a function of temperature and of pH. The experiments have been performed in triplicate.

Temperatures (°C)	Half-Life (t_1/2_, h)	pH	Half-Life (t_1/2_, h)
4	44 ± 0.1	3.0	16 ± 0.1
10	46 ± 0.3	3.5	19 ± 0.2
15	47 ± 0.2	4.0	32 ± 0.1
20	48 ± 0.6	4.5	47 ± 0.3
25	47 ± 0.2	5.0	49 ± 0.6
30	46 ± 0.4	5.5	48 ± 0.4
35	40 ± 0.2	6.0	48 ± 0.2
40	38 ± 0.2	6.5	47 ± 0.5
45	29 ± 0.2	7.0	48 ± 0.7
50	6.0 ± 0.1	7.5	47 ± 0.3
55	4.0 ± 0.2	8.0	46 ± 0.2
60	0.9 ± 0.3	8.5	44 ± 0.1
		9.0	38 ± 0.3

## Data Availability

Data generated in this study are available from the authors upon reasonable request.

## Supplementary Materials

The following supporting information can be downloaded at: https://www.mdpi.com/article/10.3390/molecules28145327/s1, Figure S1: Schematic drawing of the hydrogen bonding interactions between DMA and the acarbose-derived heptasaccharide product and electron density of the latter; Figure S2: Michaelis–Menten curve of starch hydrolysis; Figure S3: Catalytic activity of native and deletion mutant α-amylases (DMA and PPA); Figure S4: Superposition of TMA onto DMA; Figure S5: Ion binding in five α-amylases; Figure S6: Hydrogen-bonding in five α-amylases; Figure S7: Salt-bridge networks in five α-amylases; Figure S8: Surface salt-bridges in five α-amylases; Figure S9: Hydrophobic clusters in five α-amylases; Figure S10: Surface presentations of TMA/α-AI and DMA/α-AI complexes; Figure S11: Interacting residues in TMA/α-AI and DMA/α-AI complexes; Figure S12: Alignment of 129 insect α-amylases.; Figure S13: Alignment of variable amino acid sites in the *Drosophila melanogaster* species subgroup; Figure S14: Maximum-likelihood tree of 240 insect α-amylase sequences; Figure S15: Unrooted Maximum-likelihood tree of 127 α-amylase sequences representing 54 dipteran species; Table S1: Ancestral state reconstruction at sites considered as positively selected in the *D. melanogaster* branch.

## Sample Availability

Samples used in this study are available from the authors upon reasonable request.
