# Supplementary material for: Structural and Functional Characterization of Drosophila melanogaster α-Amylase
Source: Molecules. 2023 Jul 11;28(14):5327. doi: 10.3390/molecules28145327 (PMC10384113; doi:10.3390/molecules28145327)
Supplement: Supplementary file 1 [file molecules-28-05327-s001.zip › molecules-2378114-Supplementary Figure S12.pdf]

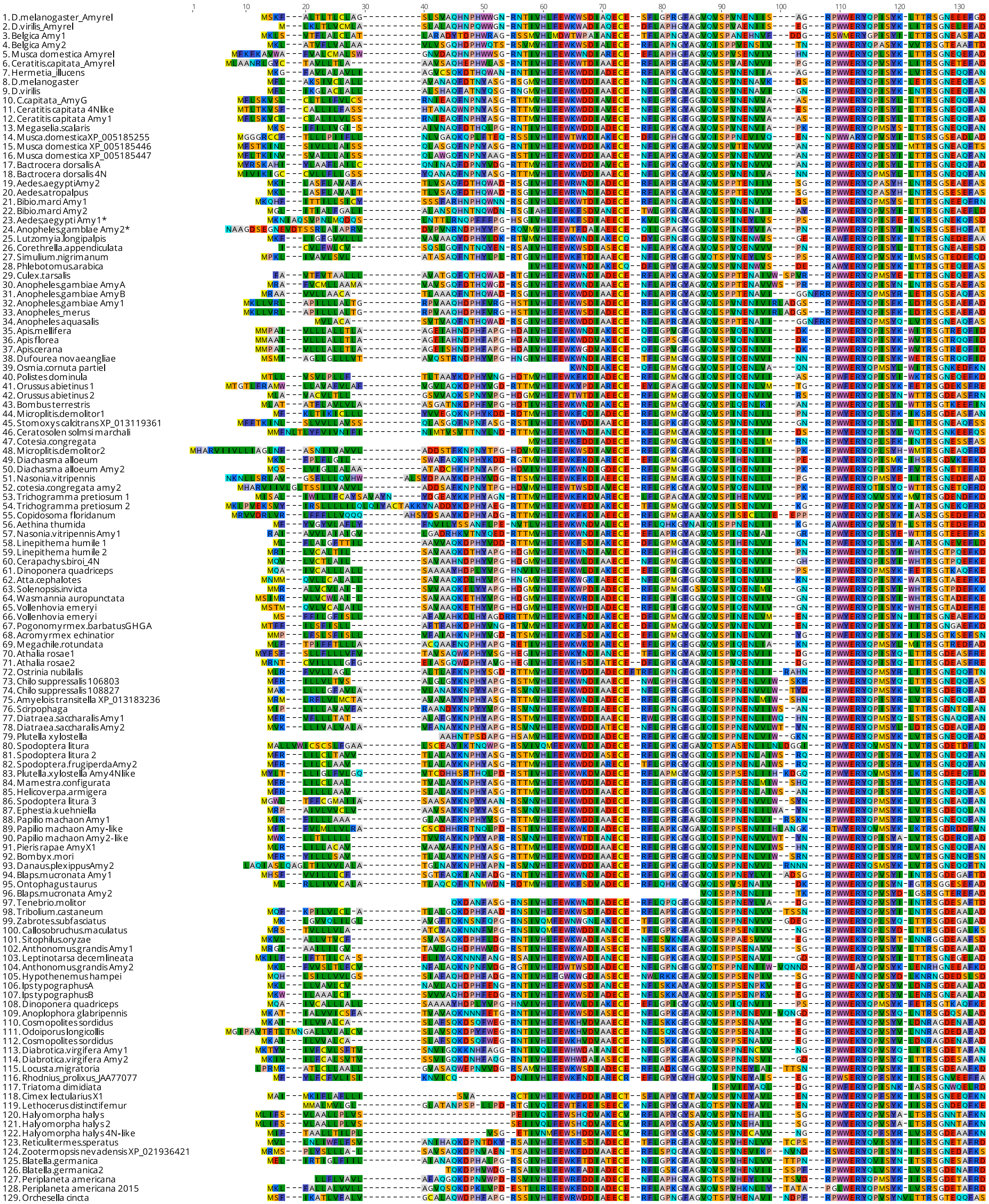

1 10 20 30 40 50 60 70 80 90 100 110 120 130
1. D.melanogaster_Amyrel
2. D.virilis_Amyrel
3. Belgica Amy1
4. Belgica Amy2
5. Musca domestica Amyrel
6. Ceratitis.capitata_Amyrel
7. Hermetia_illucens
8. D.melanogaster
9. D.virilis
10. C.capitata_AmyG
11. Ceratitis capitata 4Nlike
12. Ceratitis capitata Amy1
13. Megaselia.scalaris
14. Musca.domesticaXP_005185255
15. Musca domestica XP_005185446
16. Musca domestica XP_005185447
17. Bactrocera dorsalis A
18. Bactrocera dorsalis 4N
19. Aedes.aegyptiAmy2
20. Aedes.atropalpus
21. Bibio.marci Amy1
22. Bibio.marci Amy2
23. Aedes.aegypti Amy1*
24. Anopheles.gambiae Amy2*
25. Lutzomyia.longipalpis
26. Corethrella.appendiculata
27. Simulium.nigrimanum
28. Phlebotomus.arabica
29. Culex.tarsalis
30. Anopheles.gambiae AmyA
31. Anopheles.gambiae AmyB
32. Anopheles.gambiae Amy1
33. Anopheles_merus
34. Anopheles.aquasalis
35. Apis.mellifera
36. Apis.florea
37. Apis.cerana
38. Dufourea novaeangliae
39. Osmia.cornuta partiel
40. Polistes dominula
41. Orussus abietinus 1
42. Orussus abietinus 2
43. Bombus terrestris
44. Microplitis.demolitor1
45. Stomoxys calcitrans XP_013119361
46. Ceratosolen solmsi marchali
47. Cotesia.congregata
48. Microplitis.demolitor2
49. Diachasma alloeum
50. Diachasma alloeum Amy2
51. Nasonia.vitripennis
52. cotesia.congregata amy2
53. Trichogramma pretiosum 1
54. Trichogramma pretiosum 2
55. Copidosoma floridanum
56. Aethina thumida
57. Nasonia.vitripennis Amy1
58. Linepithema humile 1
59. Linepithema humile 2
60. Cerapachys.biroi_4N
61. Dinoponera quadriceps
62. Atta.cephalotes
63. Solenopsis.invicta
64. Wasmannia auropunctata
65. Vollenhovia emeryi
66. Vollenhovia emeryi
67. Pogonomyrmex.barbatusGHGA
68. Acromyrmex echinatior
69. Megachile.rotundata
70. Athalia rosae1
71. Athalia rosae2
72. Ostrinia nubilalis
73. Chilo suppressalis 106803
74. Chilo suppressalis 108827
75. Amyelois transitella XP_013183236
76. Scirpophaga
77. Diatraea.saccharalis Amy1
78. Diatraea.saccharalis Amy2
79. Plutella xylostella
80. Spodoptera litura
81. Spodoptera litura 2
82. Spodoptera.frugiperdaAmy2
83. Plutella.xylostella Amy4Nlike
84. Mamestra.configurata
85. Helicoverpa.armigera
86. Spodoptera litura 3
87. Ephestia.kuehniella
88. Papilio machaon Amy1
89. Papilio machaon Amy-like
90. Papilio machaon Amy2-like
91. Pieris rapae AmyX1
92. Bombyx.mori
93. Danaus.plexippusAmy2
94. Blaps.mucronata Amy1
95. Ontophagus taurus
96. Blaps.mucronata Amy2
97. Tenebrio.molitor
98. Tribolium.castaneum
99. Zabrotes.subfasciatus
100. Callosobruchus.maculatus
101. Sitophilus.oryzae
102. Anthonomus.grandis Amy1
103. Leptinotarsa decemlineata
104. Anthonomus.grandis Amy2
105. Hypothenemus hampei
106. Ips typographusA
107. Ips typographusB
108. Dinoponera quadriceps
109. Anoplophora glabripennis
110. Cosmopolites sordidus
111. Odoiporus longicollis
112. Cosmopolites sordidus
113. Diabrotica.virgifera Amy1
114. Diabrotica.virgifera Amy2
115. Locusta.migratoria
116. Rhodnius_prolixus_JAA77077
117. Triatoma dimidiata
118. Cimex lectularius X1
119. Lethocerus distinctifemur
120. Halyomorpha halys
121. Halyomorpha halys2
122. Halyomorpha halys 4N-like
123. Reticulitermes.speratus
124. Zootermopsis nevadensis XP_021936421
125. Blatella.germanica
126. Blatella.germanica2
127. Periplaneta americana
128. Periplaneta americana 2015
129. Orchesella cincta

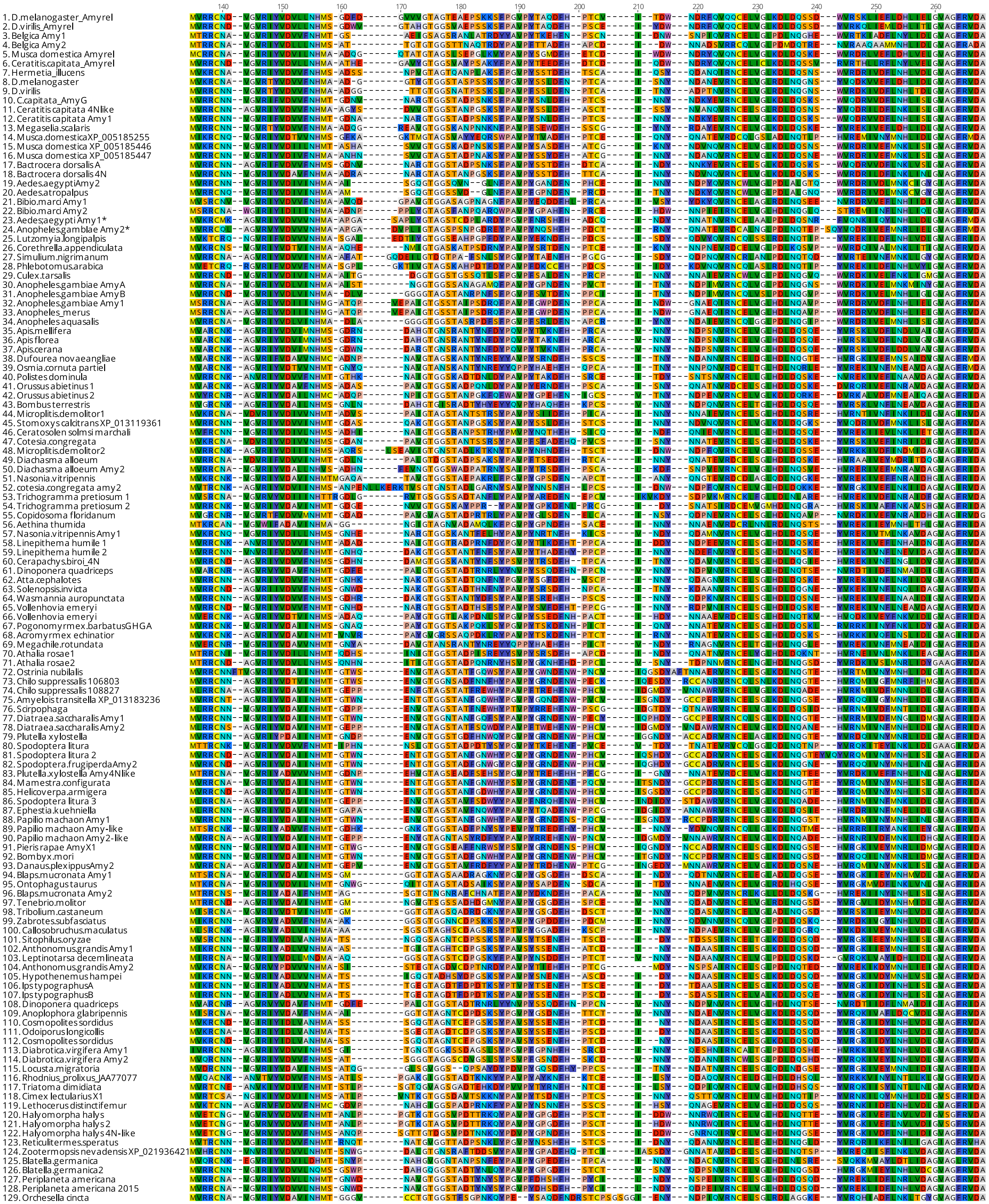
140
150
160
170
180
190
200
210
220
230
240
250
260
1. D.melanogaster_Amyrel
2. D.virilis_Amyrel
3. Belgica Amy1
4. Belgica Amy2
5. Musca domestica Amyrel
6. Ceratitis.capitata_Amyrel
7. Hermetia_illucens
8. D.melanogaster
9. D.virilis
10. C.capitata_AmyG
11. Ceratitis capitata 4Nlike
12. Ceratitis capitata Amy1
13. Megaselia.scalaris
14. Musca.domesticaXP_005185255
15. Musca domestica XP_005185446
16. Musca domestica XP_005185447
17. Bactrocera dorsalis A
18. Bactrocera dorsalis 4N
19. Aedes.aegyptiAmy2
20. Aedes.atropalpus
21. Bibio.marci Amy1
22. Bibio.marci Amy2
23. Aedes.aegypti Amy1*
24. Anopheles.gamblae Amy2*
25. Lutzomyia.longipalpis
26. Corethrella.appendiculata
27. Simulium.nigrimanum
28. Phlebotomus.arabica
29. Culex.tarsalis
30. Anopheles.gambiae AmyA
31. Anopheles.gambiae AmyB
32. Anopheles.gambiae Amy1
33. Anopheles_merus
34. Anopheles.aquasalis
35. Apis.mellifera
36. Apis.florea
37. Apis.cerana
38. Dufourea novaeangliae
39. Osmia.cornuta partiel
40. Polistes dominula
41. Orussus abietinus 1
42. Orussus abietinus 2
43. Bombus terrestris
44. Microplitis.demolitor1
45. Stomoxys calcitrans XP_013119361
46. Ceratosolen solmsi marchali
47. Cotesia.congregata
48. Microplitis.demolitor2
49. Diachasma alloeum
50. Diachasma alloeum Amy2
51. Nasonia.vitripennis
52. cotesia.congregata amy2
53. Trichogramma pretiosum 1
54. Trichogramma pretiosum 2
55. Copidosoma floridanum
56. Aethina thumida
57. Nasonia.vitripennis Amy1
58. Linepithema humile 1
59. Linepithema humile 2
60. Cerapachys.biroi_4N
61. Dinoponera quadriceps
62. Atta.cephalotes
63. Solenopsis.invicta
64. Wasmannia auropunctata
65. Vollenhovia emeryi
66. Vollenhovia emeryi
67. Pogonomyrmex.barbatusGHGA
68. Acromyrmex echinatior
69. Megachile.rotundata
70. Athalia rosae1
71. Athalia rosae2
72. Ostrinia nubilalis
73. Chilo suppressalis 106803
74. Chilo suppressalis 108827
75. Amyelois transitella XP_013183236
76. Scirpophaga
77. Diatraea.saccharalis Amy1
78. Diatraea.saccharalis Amy2
79. Plutella xylostella
80. Spodoptera litura
81. Spodoptera litura 2
82. Spodoptera.frugiperdaAmy2
83. Plutella.xylostella Amy4Nlike
84. Mamestra.configurata
85. Helicoverpa.armigera
86. Spodoptera litura 3
87. Ephestia.kuehniella
88. Papilio machaon Amy1
89. Papilio machaon Amy-like
90. Papilio machaon Amy2-like
91. Pieris rapae AmyX1
92. Bombyx.mori
93. Danaus.plexippusAmy2
94. Blaps.mucronata Amy1
95. Ontophagus taurus
96. Blaps.mucronata Amy2
97. Tenebrio.molitor
98. Tribolium.castaneum
99. Zabrotes.subfasciatus
100. Callosobruchus.maculatus
101. Sitophilus.oryzae
102. Anthonomus.grandis Amy1
103. Leptinotarsa decemlineata
104. Anthonomus.grandis Amy2
105. Hypothenemus hampei
106. Ips typographusA
107. Ips typographusB
108. Dinoponera quadriceps
109. Anoplophora glabripennis
110. Cosmopolites sordidus
111. Odoiporus longicollis
112. Cosmopolites sordidus
113. Diabrotica.virgifera Amy1
114. Diabrotica.virgifera Amy2
115. Locusta.migratoria
116. Rhodnius_prolixus_JAA77077
117. Triatoma dimidiata
118. Cimex lectularius X1
119. Lethocerus distinctifemur
120. Halyomorpha halys
121. Halyomorpha halys2
122. Halyomorpha halys 4N-like
123. Reticulitermes.speratus
124. Zootermopsis nevadensis XP_021936421
125. Blatella.germanica
126. Blatella.germanica2
127. Periplaneta americana
128. Periplaneta americana 2015
129. Orchesella cincta

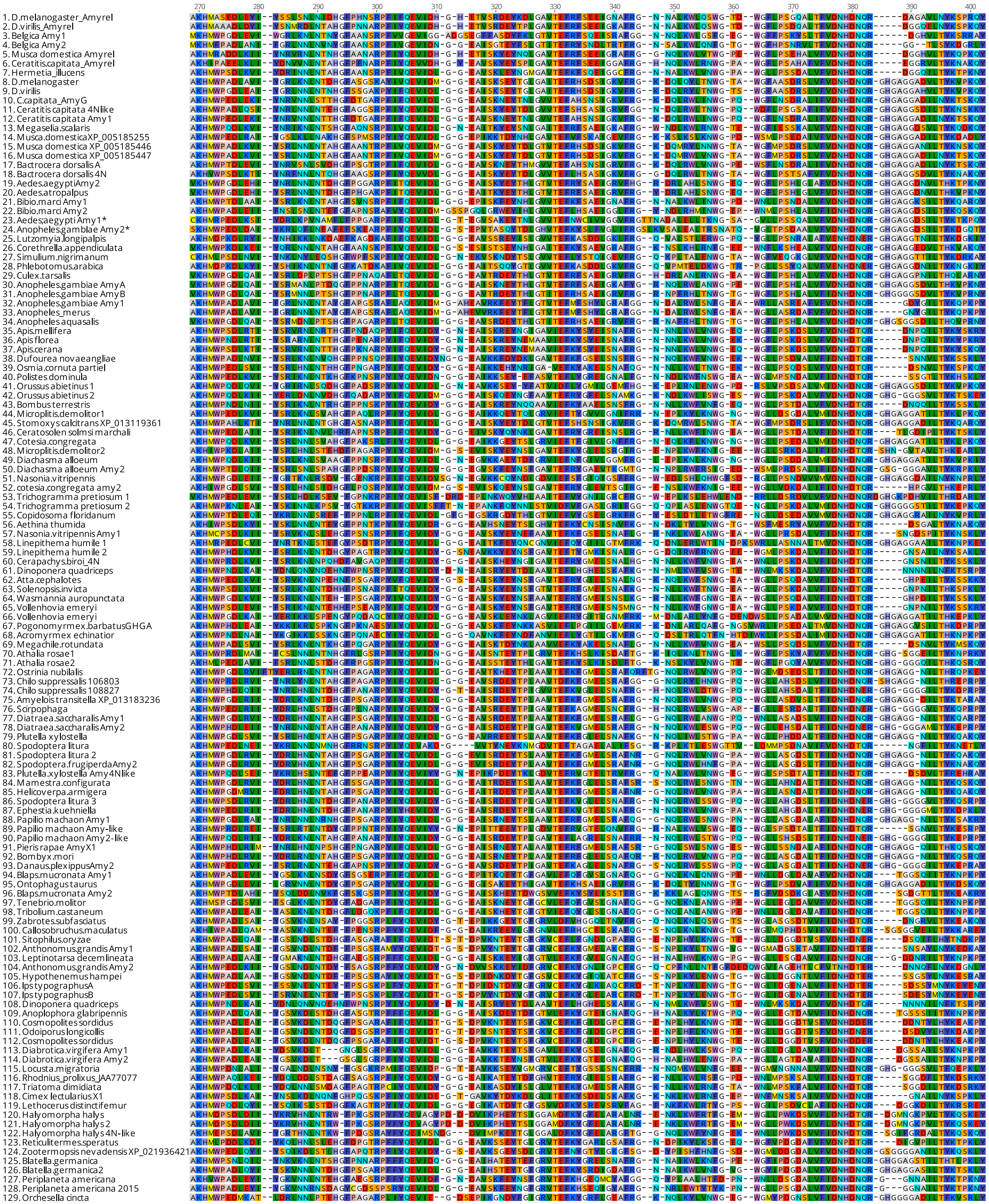

270 280 290 300 310 320 330 340 350 360 370 380 390 400
1. D.melanogaster_Amyrel
2. D.virilis_Amyrel
3. Belgica Amy1
4. Belgica Amy2
5. Musca domestica Amyrel
6. Ceratitis.capitata_Amyrel
7. Hermetia_illucens
8. D.melanogaster
9. D.virilis
10. C.capitata_AmyG
11. Ceratitis capitata 4Nlike
12. Ceratitis capitata Amy1
13. Megaselia.scalaris
14. Musca.domesticaXP_005185255
15. Musca domestica XP_005185446
16. Musca domestica XP_005185447
17. Bactrocera dorsalis A
18. Bactrocera dorsalis 4N
19. Aedes.aegyptiAmy2
20. Aedes.atropalpus
21. Bibio.marci Amy1
22. Bibio.marci Amy2
23. Aedes.aegypti Amy1*
24. Anopheles.gambiae Amy2*
25. Lutzomyia.longipalpis
26. Corethrella.appendiculata
27. Simulium.nigrimanum
28. Phlebotomus.arabica
29. Culex.tarsalis
30. Anopheles.gambiae AmyA
31. Anopheles.gambiae AmyB
32. Anopheles.gambiae Amy1
33. Anopheles_merus
34. Anopheles.aquasalis
35. Apis.mellifera
36. Apis.florea
37. Apis.cerana
38. Dufourea novaeangliae
39. Osmia.cornuta partiel
40. Polistes dominula
41. Orussus abietinus 1
42. Orussus abietinus 2
43. Bombus terrestris
44. Microplitis.demolitor1
45. Stomoxys calcitrans XP_013119361
46. Ceratosolen solmsi marchali
47. Cotesia.congregata
48. Microplitis.demolitor2
49. Diachasma alloeum
50. Diachasma alloeum Amy2
51. Nasonia.vitripennis
52. cotesia.congregata amy2
53. Trichogramma pretiosum 1
54. Trichogramma pretiosum 2
55. Copidosoma floridanum
56. Aethina thumida
57. Nasonia.vitripennis Amy1
58. Linepithema humile 1
59. Linepithema humile 2
60. Cerapachys.biroi_4N
61. Dinoponera quadriceps
62. Atta.cephalotes
63. Solenopsis.invicta
64. Wasmannia auropunctata
65. Vollenhovia emeryi
66. Vollenhovia emeryi
67. Pogonomyrmex.barbatusGHGA
68. Acromyrmex echinatior
69. Megachile.rotundata
70. Athalia rosae1
71. Athalia rosae2
72. Ostrinia nubilalis
73. Chilo suppressalis 106803
74. Chilo suppressalis 108827
75. Amyelois transitella XP_013183236
76. Scirpophaga
77. Diatraea.saccharalis Amy1
78. Diatraea.saccharalis Amy2
79. Plutella xylostella
80. Spodoptera litura
81. Spodoptera litura 2
82. Spodoptera.frugiperdaAmy2
83. Plutella.xylostella Amy4Nlike
84. Mamestra.configurata
85. Helicoverpa.armigera
86. Spodoptera litura 3
87. Ephestia.kuehniella
88. Papilio machaon Amy1
89. Papilio machaon Amy-like
90. Papilio machaon Amy2-like
91. Pieris rapae AmyX1
92. Bombyx.mori
93. Danaus.plexippusAmy2
94. Blaps.mucronata Amy1
95. Ontophagus taurus
96. Blaps.mucronata Amy2
97. Tenebrio.molitor
98. Tribolium.castaneum
99. Zabrotes.subfasciatus
100. Callosobruchus.maculatus
101. Sitophilus.oryzae
102. Anthonomus.grandis Amy1
103. Leptinotarsa decemlineata
104. Anthonomus.grandis Amy2
105. Hypothenemus hampei
106. Ips typographusA
107. Ips typographusB
108. Dinoponera quadriceps
109. Anoplophora glabripennis
110. Cosmopolites sordidus
111. Odoiporus longicollis
112. Cosmopolites sordidus
113. Diabrotica.virgifera Amy1
114. Diabrotica.virgifera Amy2
115. Locusta.migratoria
116. Rhodnius_prolixus_JAA77077
117. Triatoma dimidiata
118. Cimex lectularius X1
119. Lethocerus distinctifemur
120. Halyomorpha halys
121. Halyomorpha halys 2
122. Halyomorpha halys 4N-like
123. Reticulitermes.speratus
124. Zootermopsis nevadensis XP_021936421
125. Blatella.germanica
126. Blatella.germanica2
127. Periplaneta americana
128. Periplaneta americana 2015
129. Orchesella cincta

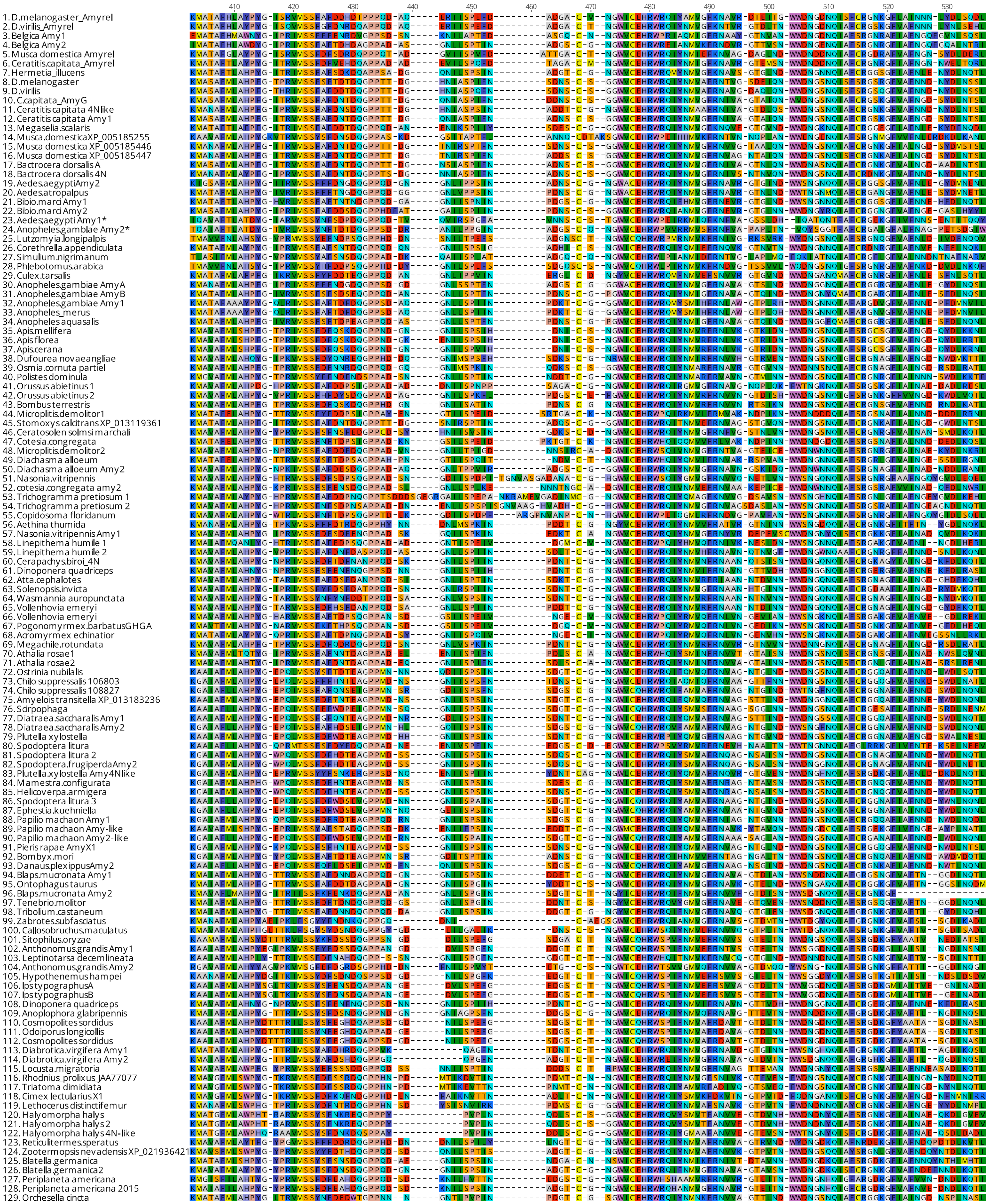
410
420
430
440
450
460
470
480
490
500
510
520
530
1. D.melanogaster_Amyrel
2. D.virilis_Amyrel
3. Belgica Amy1
4. Belgica Amy2
5. Musca domestica Amyrel
6. Ceratitis.capitata_Amyrel
7. Hermetia_illucens
8. D.melanogaster
9. D.virilis
10. C.capitata_AmyG
11. Ceratitis capitata 4Nlike
12. Ceratitis capitata Amy1
13. Megaselia.scalaris
14. Musca.domesticaXP_005185255
15. Musca domestica XP_005185446
16. Musca domestica XP_005185447
17. Bactrocera dorsalis A
18. Bactrocera dorsalis 4N
19. Aedes.aegyptiAmy2
20. Aedes.atropalpus
21. Bibio.marci Amy1
22. Bibio.marci Amy2
23. Aedes.aegypti Amy1*
24. Anopheles.gambiae Amy2*
25. Lutzomyia.longipalpis
26. Corethrella.appendiculata
27. Simulium.nigrimanum
28. Phlebotomus.arabica
29. Culex.tarsalis
30. Anopheles.gambiae AmyA
31. Anopheles.gambiae AmyB
32. Anopheles.gambiae Amy1
33. Anopheles_merus
34. Anopheles.aquasalis
35. Apis.mellifera
36. Apis.florea
37. Apis.cerana
38. Dufourea novaeangliae
39. Osmia.cornuta partiel
40. Polistes dominula
41. Orussus abietinus 1
42. Orussus abietinus 2
43. Bombus terrestris
44. Microplitis.demolitor1
45. Stomoxys calcitrans XP_013119361
46. Ceratosolen solmsi marchali
47. Cotesia.congregata
48. Microplitis.demolitor2
49. Diachasma alloeum
50. Diachasma alloeum Amy2
51. Nasonia.vitripennis
52. cotesia.congregata amy2
53. Trichogramma pretiosum 1
54. Trichogramma pretiosum 2
55. Copidosoma floridanum
56. Aethina thumida
57. Nasonia.vitripennis Amy1
58. Linepithema humile 1
59. Linepithema humile 2
60. Cerapachys.biroi_4N
61. Dinoponera quadriceps
62. Atta.cephalotes
63. Solenopsis.invicta
64. Wasmannia auropunctata
65. Vollenhovia emeryi
66. Vollenhovia emeryi
67. Pogonomyrmex.barbatusGHGA
68. Acromyrmex echinatior
69. Megachile.rotundata
70. Athalia rosae1
71. Athalia rosae2
72. Ostrinia nubilalis
73. Chilo suppressalis 106803
74. Chilo suppressalis 108827
75. Amyelois transitella XP_013183236
76. Scirpophaga
77. Diatraea.saccharalis Amy1
78. Diatraea.saccharalis Amy2
79. Plutella xylostella
80. Spodoptera litura
81. Spodoptera litura 2
82. Spodoptera.frugiperdaAmy2
83. Plutella.xylostella Amy4Nlike
84. Mamestra.configurata
85. Helicoverpa.armigera
86. Spodoptera litura 3
87. Ephestia.kuehniella
88. Papilio machaon Amy1
89. Papilio machaon Amy-like
90. Papilio machaon Amy2-like
91. Pieris rapae AmyX1
92. Bombyx.mori
93. Danaus.plexippusAmy2
94. Blaps.mucronata Amy1
95. Ontophagus taurus
96. Blaps.mucronata Amy2
97. Tenebrio.molitor
98. Tribolium.castaneum
99. Zabrotes.subfasciatus
100. Callosobruchus.maculatus
101. Sitophilus.oryzae
102. Anthonomus.grandis Amy1
103. Leptinotarsa decemlineata
104. Anthonomus.grandis Amy2
105. Hypothenemus hampei
106. Ips typographusA
107. Ips typographusB
108. Dinoponera quadriceps
109. Anoplophora glabripennis
110. Cosmopolites sordidus
111. Odoiporus longicollis
112. Cosmopolites sordidus
113. Diabrotica.virgifera Amy1
114. Diabrotica.virgifera Amy2
115. Locusta.migratoria
116. Rhodnius_prolixus_JAA77077
117. Triatoma dimidiata
118. Cimex lectularius X1
119. Lethocerus distinctifemur
120. Halyomorpha halys
121. Halyomorpha halys2
122. Halyomorpha halys 4N-like
123. Reticulitermes.speratus
124. Zootermopsis nevadensis XP_021936421
125. Blatella.germanica
126. Blatella.germanica2
127. Periplaneta americana
128. Periplaneta americana 2015
129. Orchesella cincta

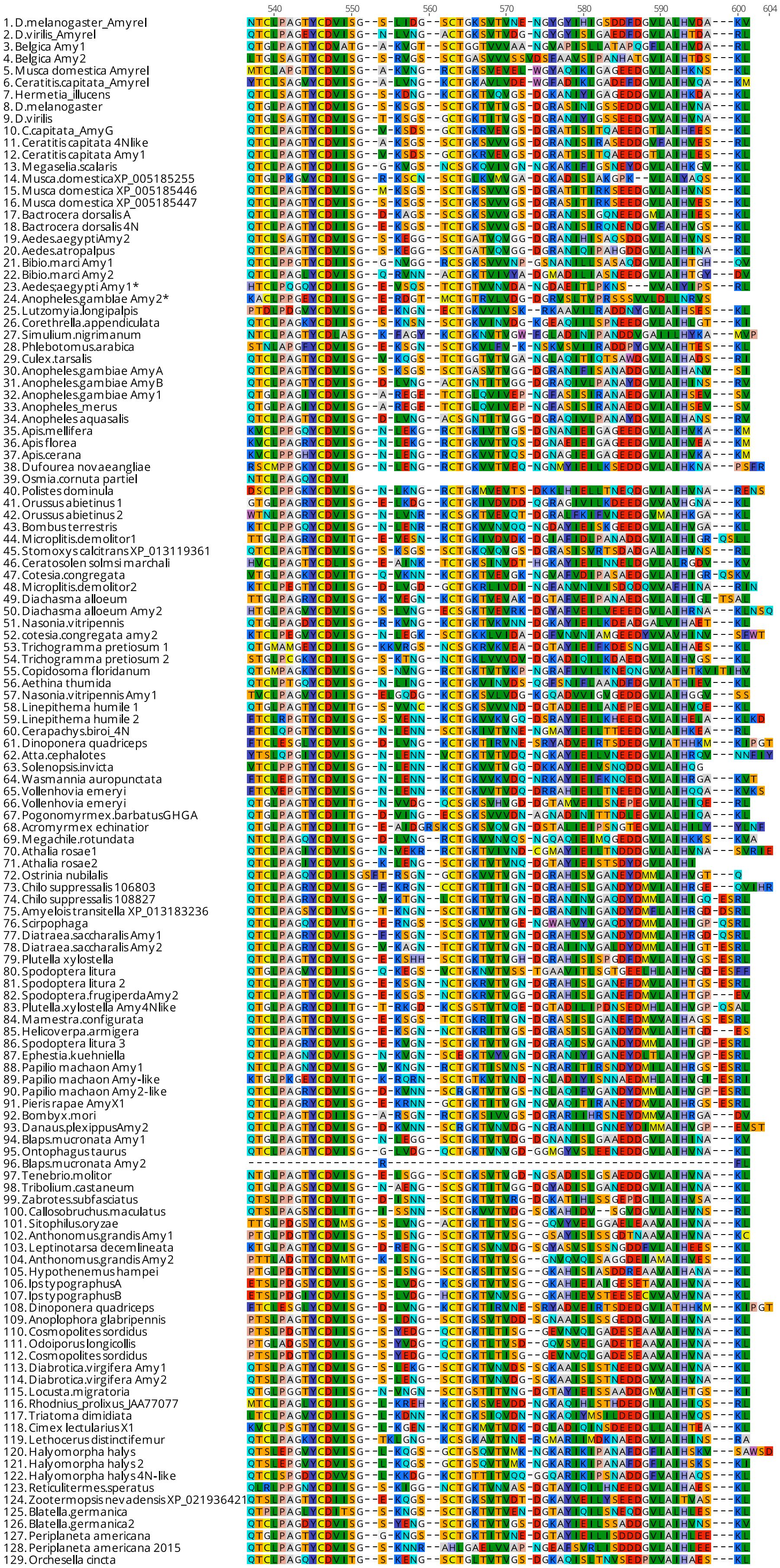

540
550
560
570
580
590
600
604
1. D.melanogaster_Amyrel
2. D.virilis_Amyrel
3. Belgica Amy1
4. Belgica Amy2
5. Musca domestica Amyrel
6. Ceratitis.capitata_Amyrel
7. Hermetia_illucens
8. D.melanogaster
9. D.virilis
10. C.capitata_AmyG
11. Ceratitis capitata 4Nlike
12. Ceratitis capitata Amy1
13. Megaselia.scalaris
14. Musca.domesticaXP_005185255
15. Musca domestica XP_005185446
16. Musca domestica XP_005185447
17. Bactrocera dorsalis A
18. Bactrocera dorsalis 4N
19. Aedes.aegyptiAmy2
20. Aedes.atropalpus
21. Bibio.marci Amy1
22. Bibio.marci Amy2
23. Aedes.aegypti Amy1*
24. Anopheles.gamblae Amy2*
25. Lutzomyia.longipalpis
26. Corethrella.appendiculata
27. Simulium.nigrimanum
28. Phlebotomus.arabica
29. Culex.tarsalis
30. Anopheles.gambiae AmyA
31. Anopheles.gambiae AmyB
32. Anopheles.gambiae Amy1
33. Anopheles_merus
34. Anopheles aquasalis
35. Apis.mellifera
36. Apis florea
37. Apis.cerana
38. Dufourea novaeangliae
39. Osmia.cornuta partiel
40. Polistes dominula
41. Orussus abietinus 1
42. Orussus abietinus 2
43. Bombus terrestris
44. Microplitis.demolitor1
45. Stomoxys calcitrans XP_013119361
46. Ceratosolen solmsi marchali
47. Cotesia.congregata
48. Microplitis.demolitor2
49. Diachasma alloeum
50. Diachasma alloeum Amy2
51. Nasonia.vitripennis
52. cotesia.congregata amy2
53. Trichogramma pretiosum 1
54. Trichogramma pretiosum 2
55. Copidosoma floridanum
56. Aethina thumida
57. Nasonia.vitripennis Amy1
58. Linepithema humile 1
59. Linepithema humile 2
60. Cerapachys.biroi_4N
61. Dinoponera quadriceps
62. Atta.cephalotes
63. Solenopsis.invicta
64. Wasmannia auropunctata
65. Vollenhovia emeryi
66. Vollenhovia emeryi
67. Pogonomyrmex.barbatusGHGA
68. Acromyrmex echinatior
69. Megachile.rotundata
70. Athalia rosae1
71. Athalia rosae2
72. Ostrinia nubilalis
73. Chilo suppressalis 106803
74. Chilo suppressalis 108827
75. Amyelois transitella XP_013183236
76. Scirpophaga
77. Diatraea.saccharalis Amy1
78. Diatraea.saccharalis Amy2
79. Plutella xylostella
80. Spodoptera litura
81. Spodoptera litura 2
82. Spodoptera.frugiperdaAmy2
83. Plutella.xylostella Amy4Nlike
84. Mamestra.configurata
85. Helicoverpa.armigera
86. Spodoptera litura 3
87. Ephestia.kuehniella
88. Papilio machaon Amy1
89. Papilio machaon Amy-like
90. Papilio machaon Amy2-like
91. Pieris rapae AmyX1
92. Bombyx.mori
93. Danaus.plexippusAmy2
94. Blaps.mucronata Amy1
95. Ontophagus taurus
96. Blaps.mucronata Amy2
97. Tenebrio.molitor
98. Tribolium.castaneum
99. Zabrotes.subfasciatus
100. Callosobruchus.maculatus
101. Sitophilus.oryzae
102. Anthonomus.grandis Amy1
103. Leptinotarsa decemlineata
104. Anthonomus.grandis Amy2
105. Hypothenemus hampei
106. Ips typographusA
107. Ips typographusB
108. Dinoponera quadriceps
109. Anoplophora glabripennis
110. Cosmopolites sordidus
111. Odoiporus longicollis
112. Cosmopolites sordidus
113. Diabrotica.virgifera Amy1
114. Diabrotica.virgifera Amy2
115. Locusta.migratoria
116. Rhodnius_prolixus_JAA77077
117. Triatoma dimidiata
118. Cimex lectularius X1
119. Lethocerus distinctifemur
120. Halyomorpha halys
121. Halyomorpha halys2
122. Halyomorpha halys 4N-like
123. Reticulitermes.speratus
124. Zootermopsis nevadensis XP_021936421
125. Blatella.germanica
126. Blatella.germanica2
127. Periplaneta americana
128. Periplaneta americana 2015
129. Orchesella cincta
