# Supplementary material for: Structural and Functional Characterization of Drosophila melanogaster α-Amylase
Source: Molecules. 2023 Jul 11;28(14):5327. doi: 10.3390/molecules28145327 (PMC10384113; doi:10.3390/molecules28145327)
Supplement: Supplementary file 1 [file molecules-28-05327-s001.zip › molecules-2378114-supplementary-proof.pdf]

Supplementary Data

Structural and functional characterization of *Drosophila melanogaster* α-amylase

Moez Rhimi [1,†,‡], Jean-Luc Da Lage [2,‡], Richard Haser [1], Georges Feller [3] and Nushin Aghajari [1,*]

Correspondence: nushin.aghajari@ibcp.fr; Tel.: +33(0)472722633 (N.A.)

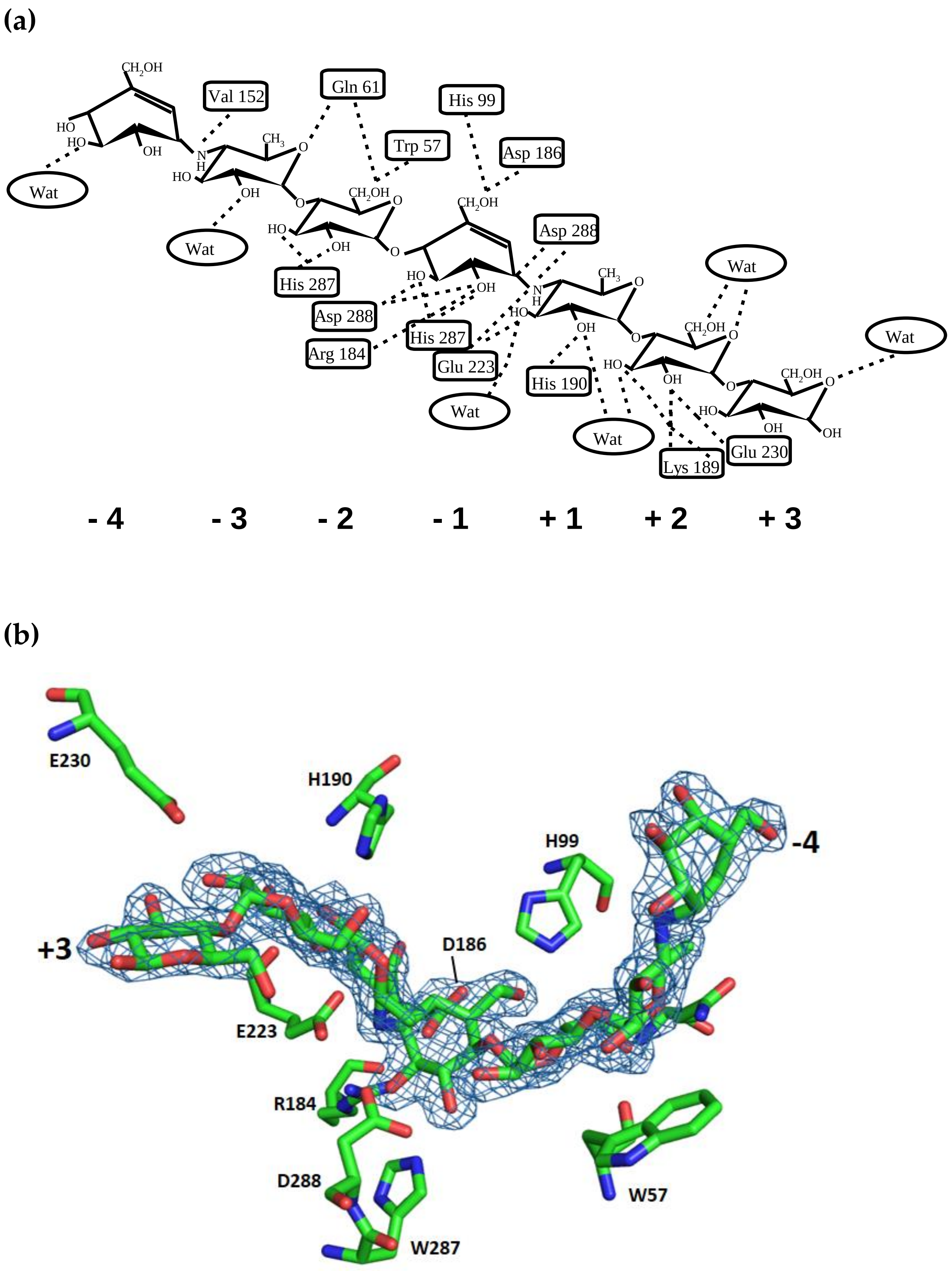

Supplementary Figure S1. (**a**) Schematic drawing of the hydrogen bonding interactions between DMA and the acarbose-derived heptasaccharide product. Under water poor conditions, DMA performs a transglycosylation reaction when in the presence of the pseudo-tetrasaccharide acarbose, and leading to a heptasaccharide occupying subsites -4 to +3. (**b**) 2Fo – Fc electron density corresponding to the heptasaccharide as observed in the crystal structure. For the ease of looking at the binding of the heptasaccharide to the active site, some of the interacting residues has not been depicted.

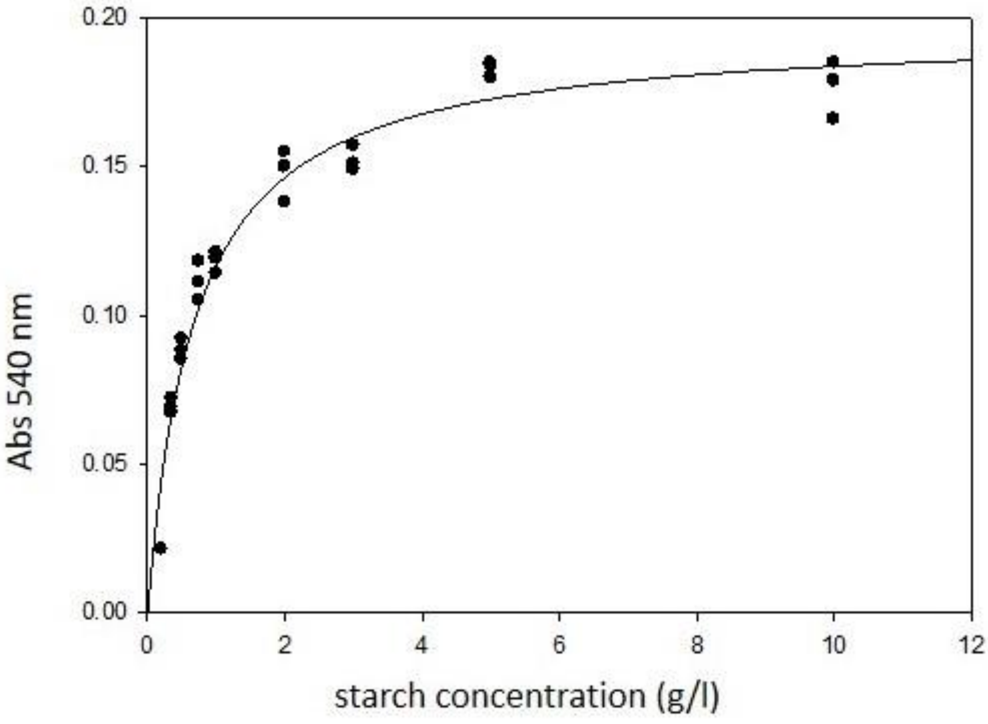

Supplementary Figure S2. Michaelis-Menten curve of DMA catalysed starch hydrolysis.

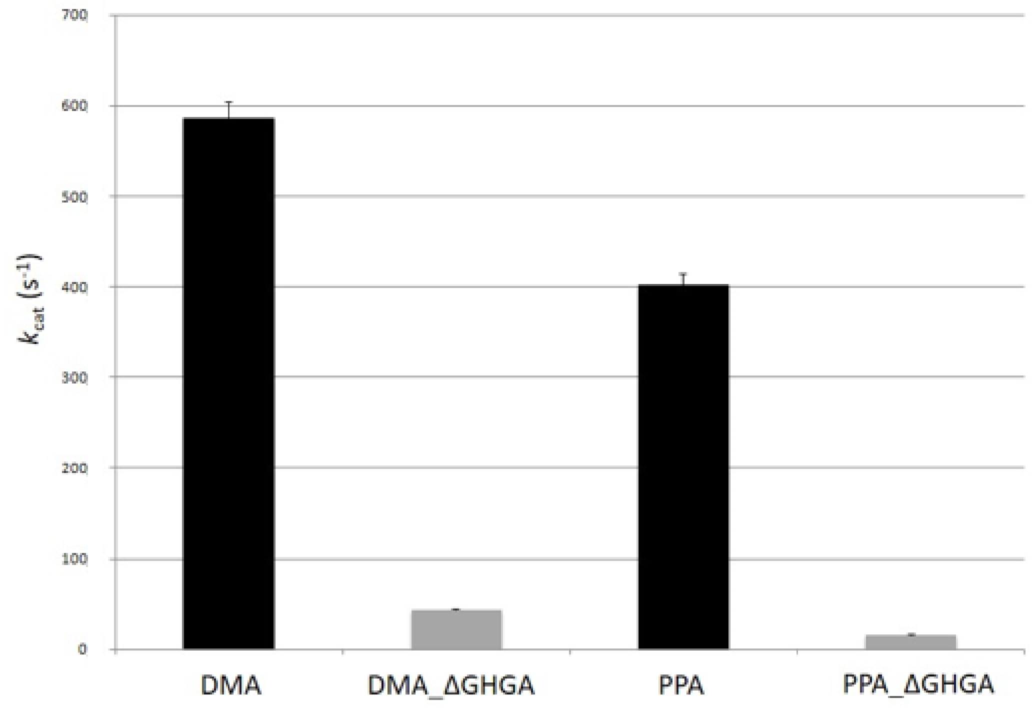

Supplementary Figure S3. Catalytic activity of native DMA compared to that of the deletion mutant DMA_ΔGHGA and of native PPA (porcine pancreatic α-amylase) compared to the deletion mutant PPA_ΔGHGA. The activity is measured on starch at 25 °C. All measurements were performed in triplicate.

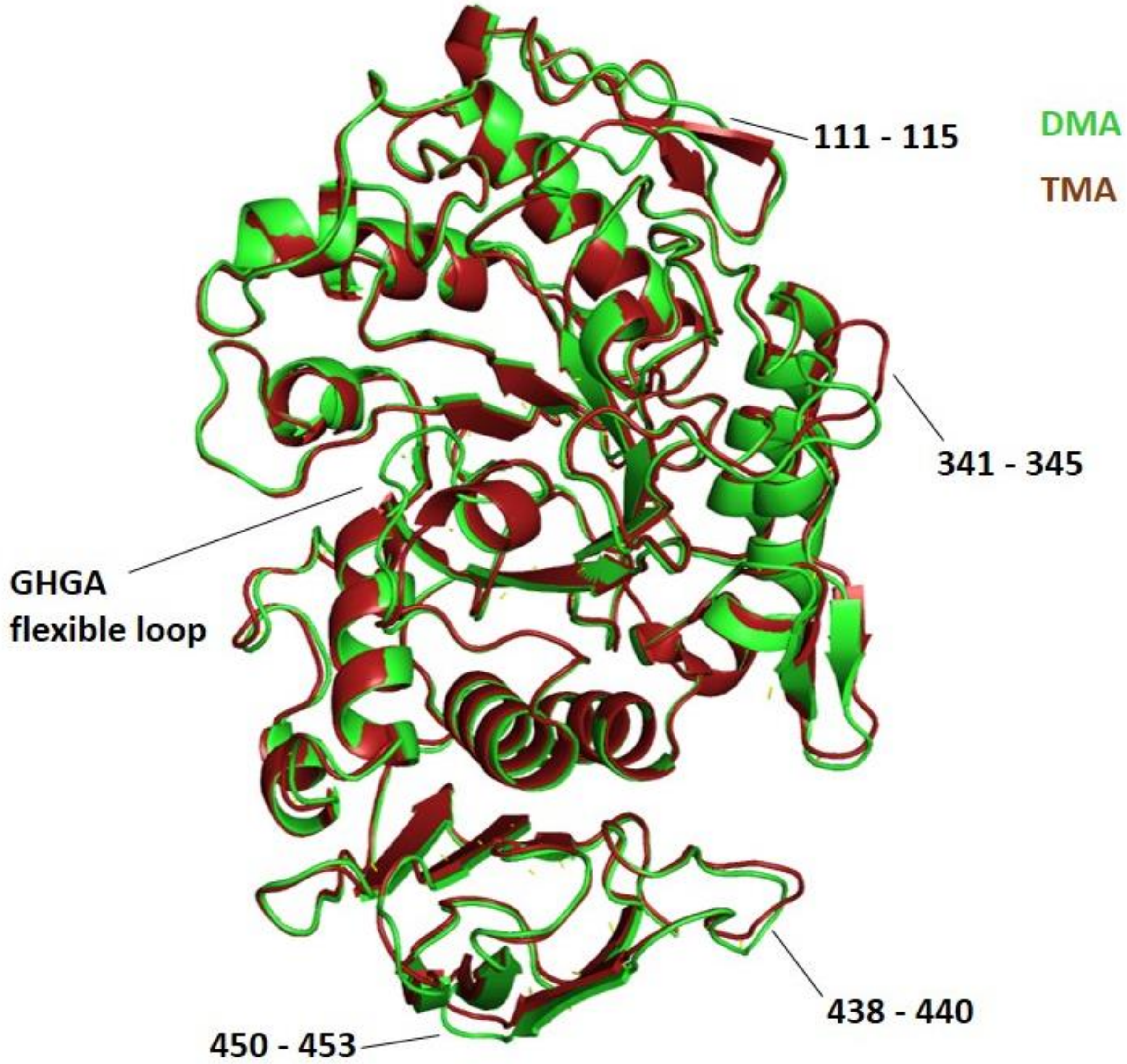

Supplementary Figure S4. Superposition of the crystal structure of *Tenebrio molitor* larvae α-amylase (brown, PDB 1JAE [34]) onto *Drosophila melanogaster* α-amylase (green, these studies) with differences in loop regions highlighted.

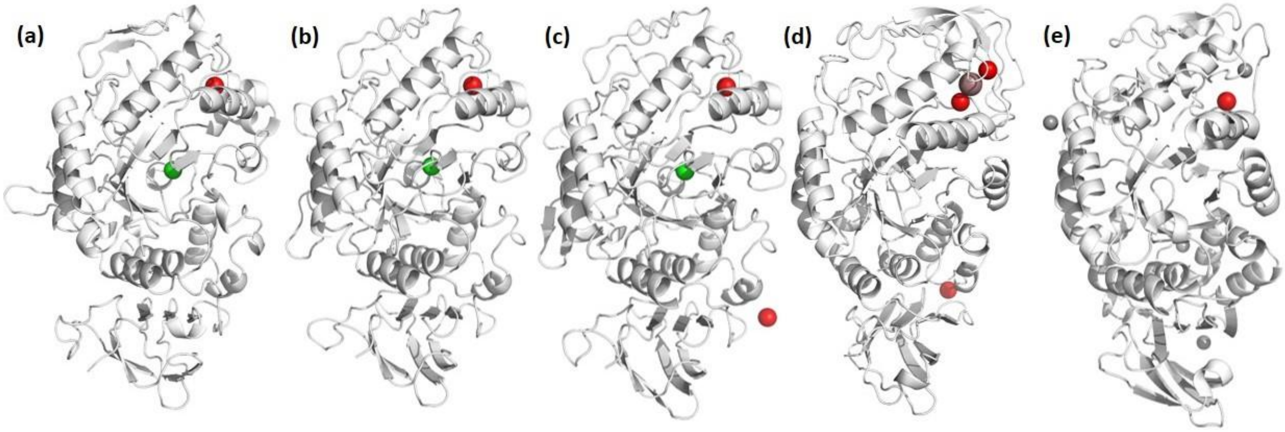

Supplementary Figure S5. Overall three-dimensional structure of a selection of α-amylases with bound ions. (**a**) the psychrophilic *Pseudoalteromonas haloplanktis* (PDB 1AQH [48]), (**b**) *Tenebrio molitor* larvae (PDB 1JAE [34]), (**c**) *Drosphila melanogaster* (these studies), (**d**) the thermostable *Bacillus licheniformis* (PDB 1BLI [71]) and (**e**) the hyperthermophilic *Pyrococcus woesei* (PDB 1MWO [72]). Calcium ions are shown as red spheres, chloride ions as green spheres and zinc ions as grey spheres.

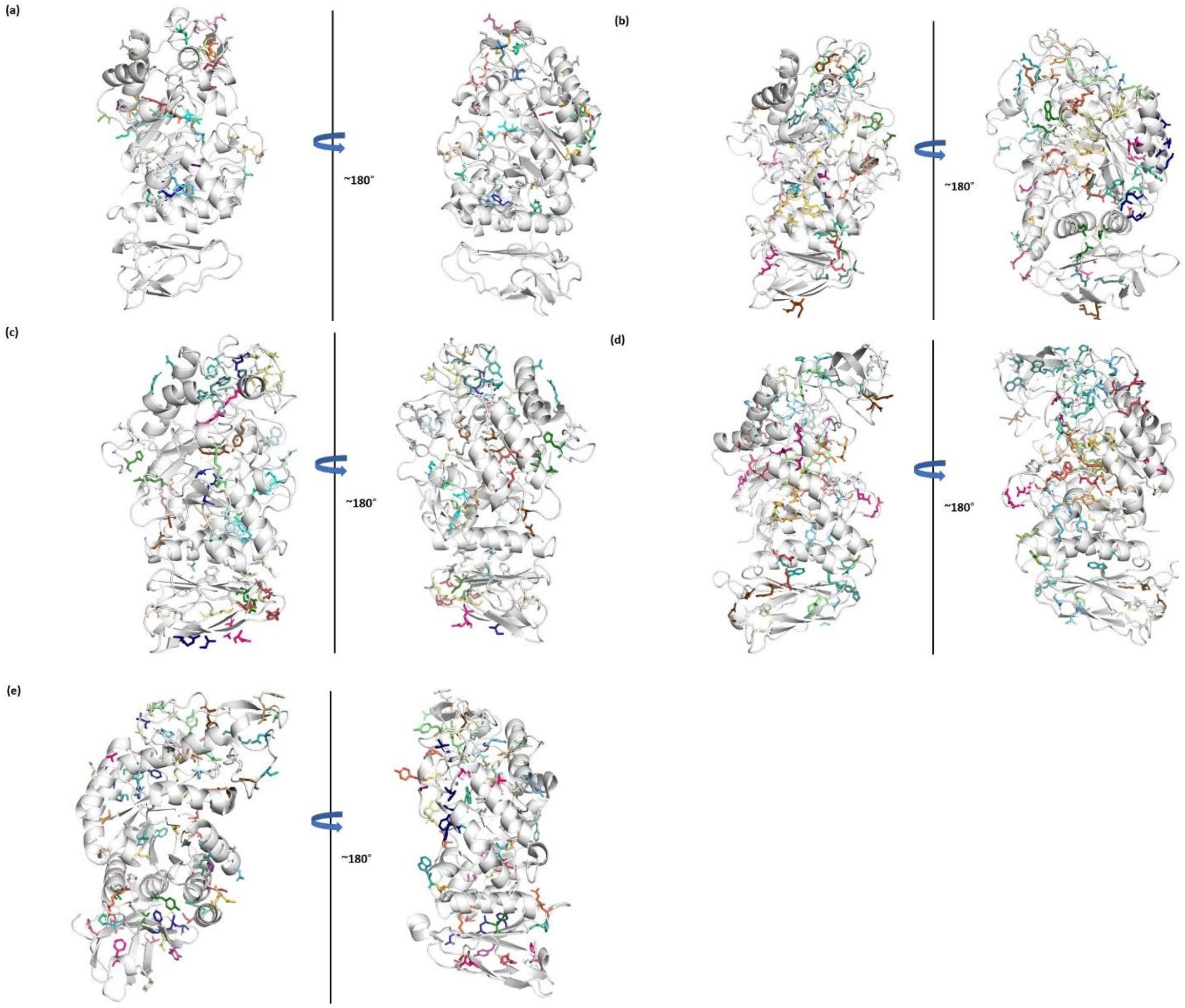

Supplementary Figure S6. Hydrogen bonding networks as calculated by "ProteinTools" [62] shown as sticks in five selected α-amylases. Different colors correspond to different hydrogen bonding networks. (**a**) The psychrophilic *Pseudoalteromonas haloplanktis* (PDB 1AQH [48]), (**b**) *Tenebrio molitor* larvae (PDB 1JAE [34]), (**c**) *Drosphila melanogaster* (these studies), (**d**) the thermostable *Bacillus licheniformis* (PDB 1BLI [71]) and (**e**) the hyperthermophilic *Pyrococcus woesei* (PDB 1MWO [72]).

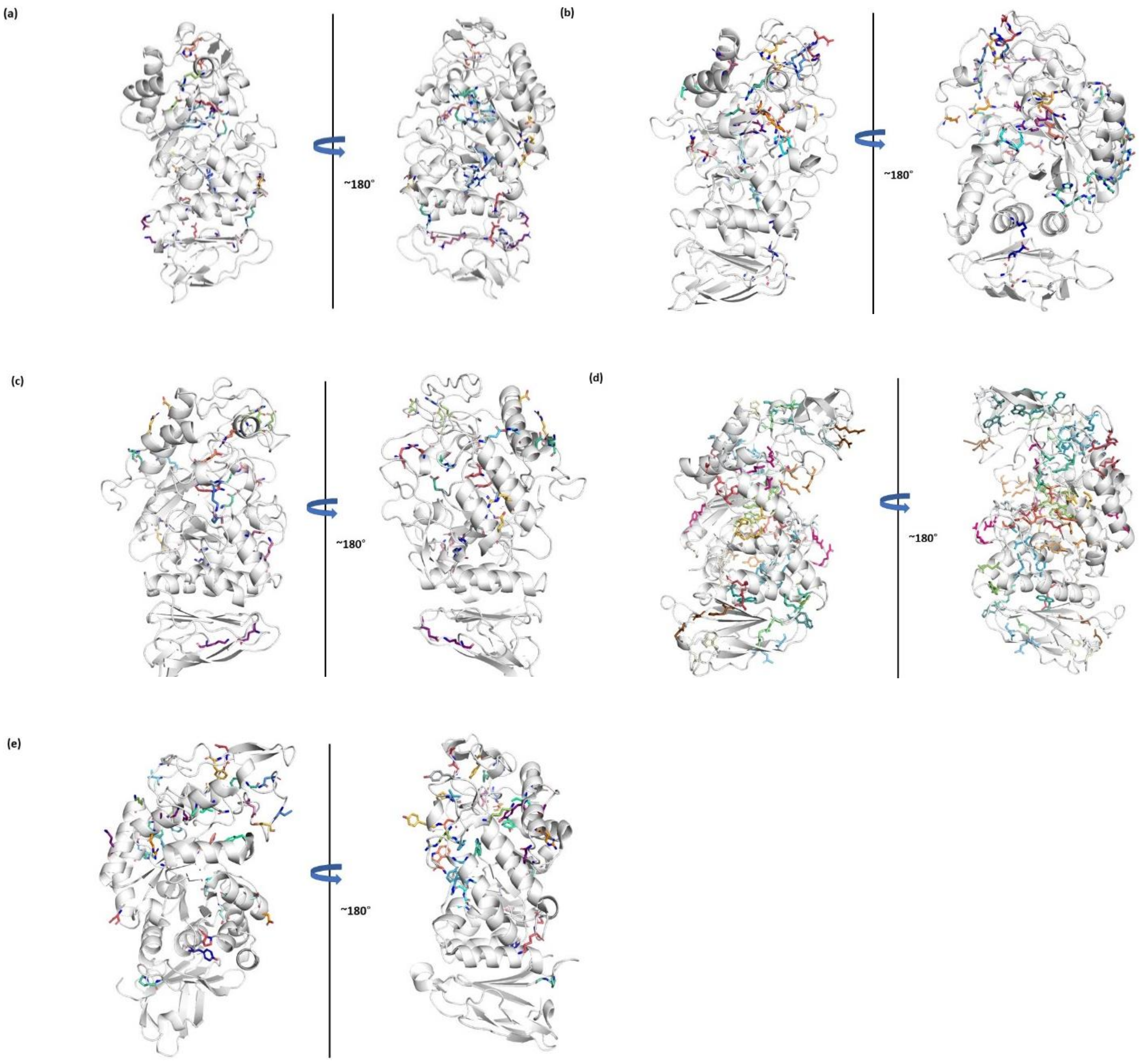

Supplementary Figure S7. Salt-bridges as calculated by "ProteinTools" [62] shown as sticks in five selected α-amylases. Different colors correspond to different salt-bridges. (**a**) The psychrophilic *Pseudoalteromonas haloplanktis* (PDB 1AQH [48]), (**b**) *Tenebrio molitor* larvae (PDB 1JAE [34]), (**c**) *Drosphila melanogaster* (these studies), (**d**) the thermostable *Bacillus licheniformis* (PDB 1BLI [71]) and (**e**) the hyperthermophilic *Pyrococcus woesei* (PDB 1MWO [72]).

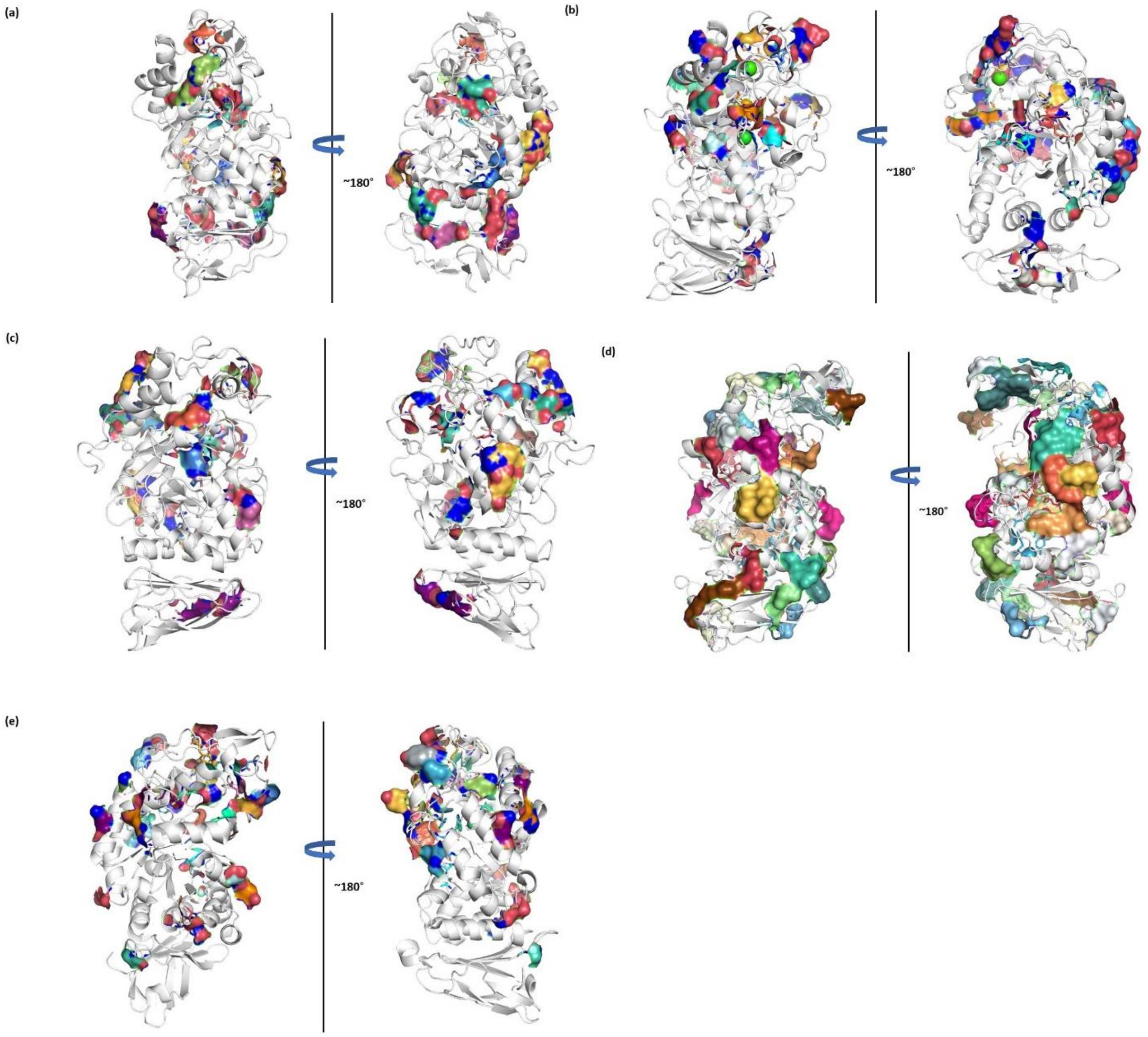

Supplementary Figure S8 Surface salt-bridges as calculated by "ProteinTools" [62] shown as surface presentation in five selected α-amylases. Different colors correspond to different salt-bridges. (**a**) The psychrophilic *Pseudoalteromonas haloplanktis* (PDB 1AQH [48]), (**b**) *Tenebrio molitor* larvae (PDB 1JAE [34]), (**c**) *Drosphila melanogaster* (these studies), (**d**) the thermostable *Bacillus licheniformis* (PDB 1BLI [71]) and (**e**) the hyperthermophilic *Pyrococcus woesei* (PDB 1MWO [72]).

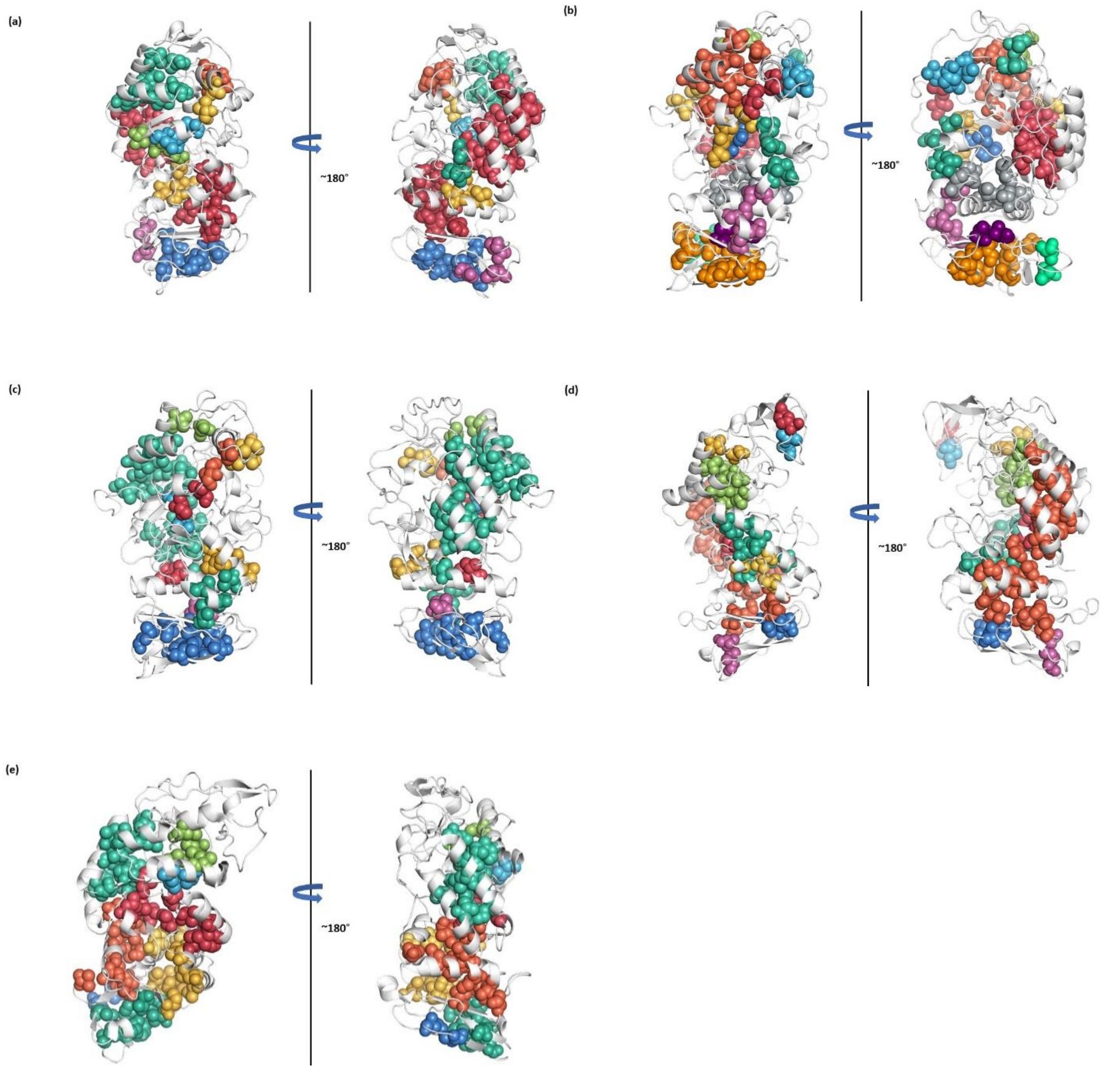

Supplementary Figure S9. Hydrophobic clusters of valines, leucine and isoleucine as calculated by "ProteinTools" [62] shown as spheres in five selected α-amylases. Different colors correspond to different clusters. (**a**) The psychrophilic *Pseudoalteromonas haloplanktis* (PDB 1AQH [48]), (**b**) *Tenebrio molitor* larvae (PDB 1JAE [34]), (**c**) *Drosphila melanogaster* (these studies), (**d**) the thermostable *Bacillus licheniformis* (PDB 1BLI [71]) and (**e**) the hyperthermophilic *Pyrococcus woesei* (PDB 1MWO [72]).

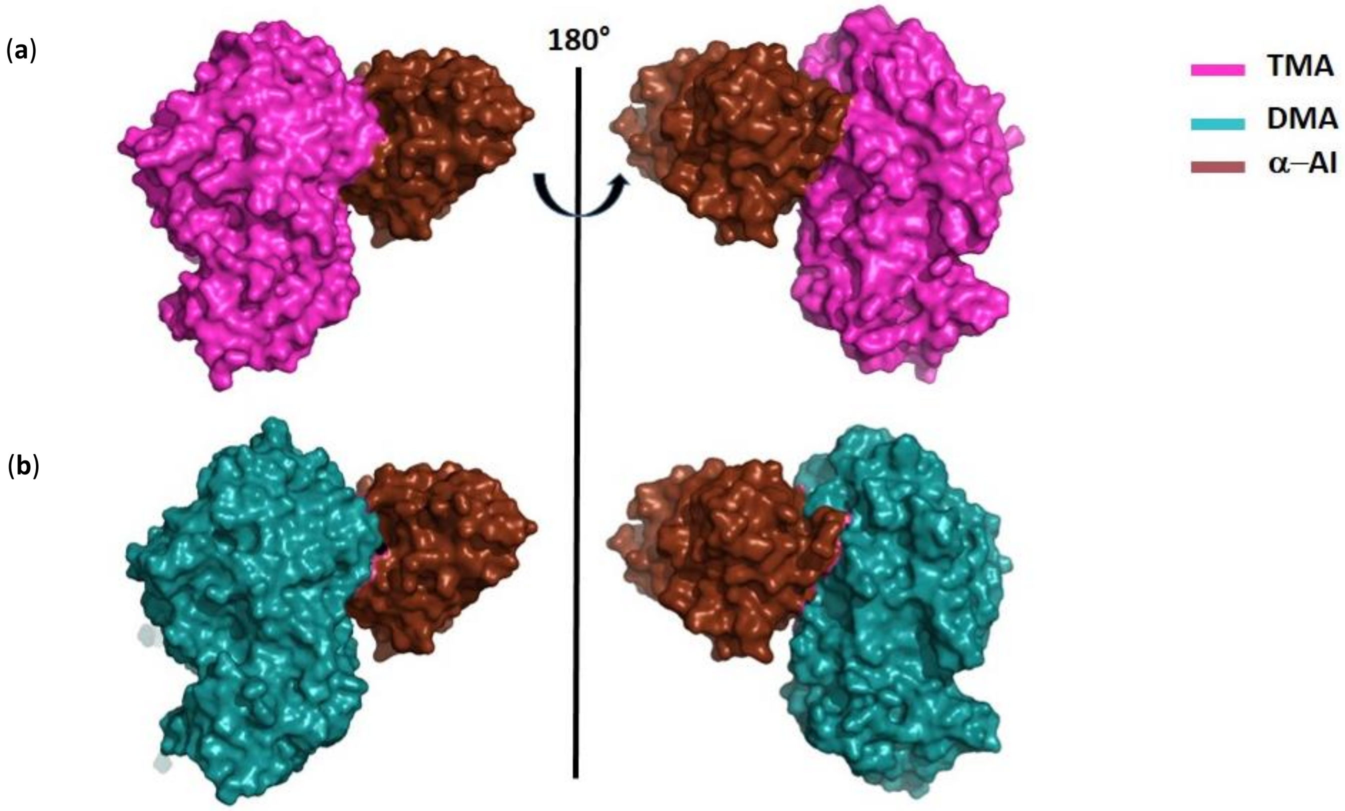

Supplementary Figure S10. Surface presentations of *P. vulgaris* α-AI complexes with (**a**) *T. molitor* α-amylase in the crystal structure of the complex [63], (**b**) from docking studies in which the three-dimensional structure of DMA has been superposed onto the structure of TMA in the TMA/α-AI complex.

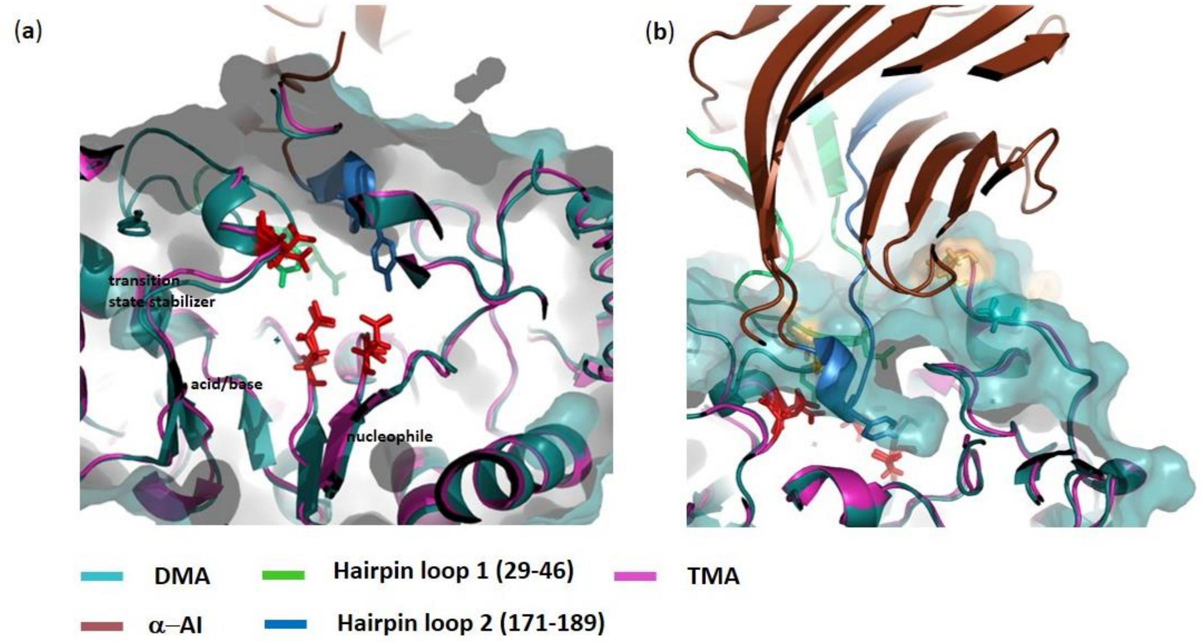

Supplementary Figure S11. Superposition of DMA onto TMA from the crystal structure of the *P. vulgaris* α-AI/TMA complex (PDB code 1VIW [63]). (**a**) Close-up on the active site showing the three essential catalytic residues (the nucleophile Asp 185/186, the general acid/base Glu 222/223, and the transition-state stabilizer Asp 287/288) of TMA/DMA, respectively, in red sticks interacting with residues from hairpin loop 1 (Tyr37, Asp38 in green) and hairpin loop 2 (Tyr186- in blue) of α-AI. (**b**) Amino-acid substitutions within the 16 other residues performing hydrogen bonds with α-AI as seen in the TMA/α-AI complex, and comprise Gln295 -> Asp, Asn137 -> Ser, Asn144 -> Asp, Ser294 -> All four highlighted in teal.

Due to the important number of pages, this figure can be uploaded as a separate file.

Supplementary Figure S12. Alignment of 129 insect α-amylases. Of the 129 sequences, 34 are of Diptera, 37 of Hymenoptera, 22 of Lepidoptera, 21 of Coleoptera, 1 Orthoptera, 7 Hemiptera, 6 Dictyoptera and 1 Collembola.

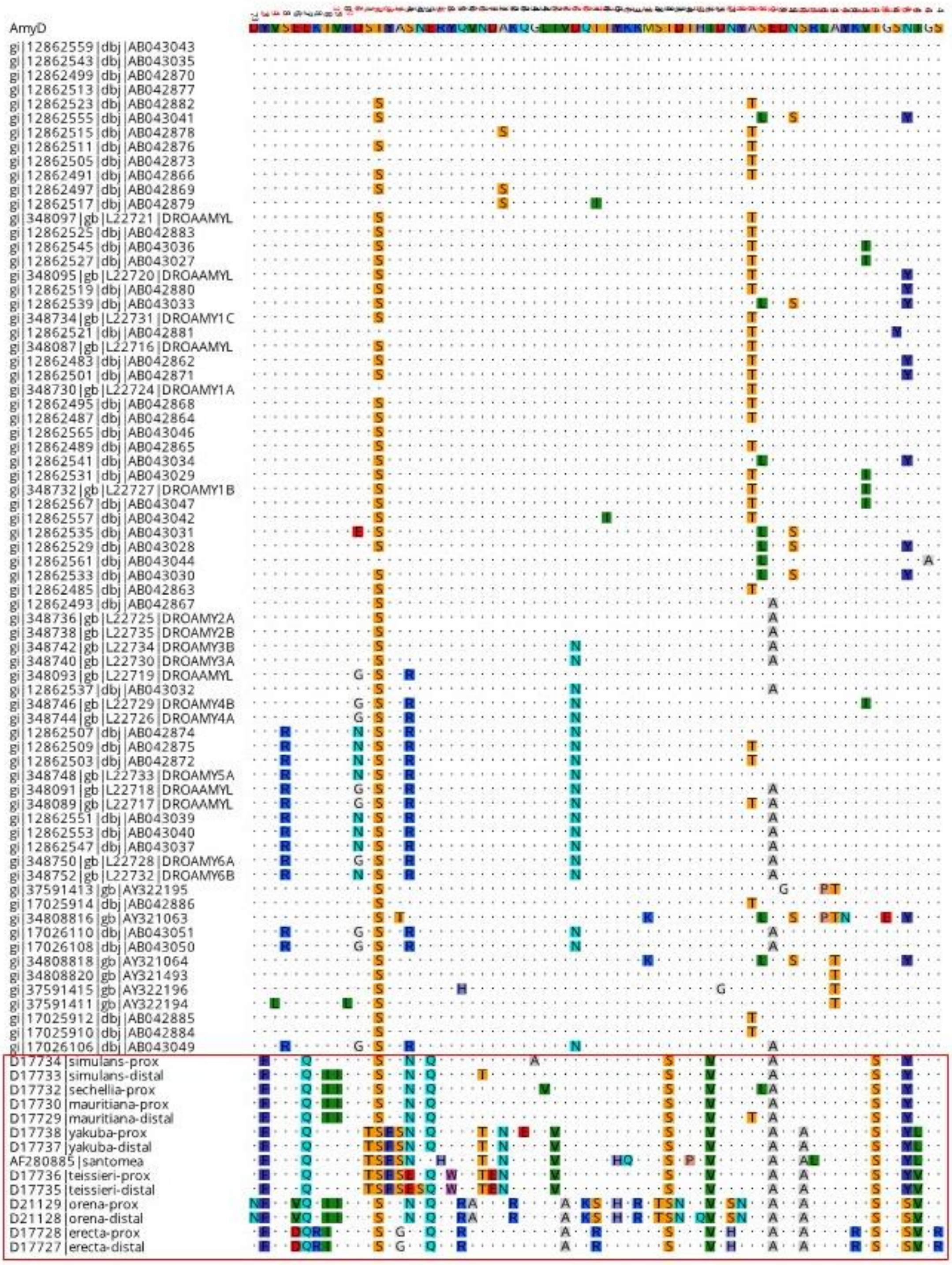

Supplementary Figure S13. Alignment of the variable amino acid sites of α-amylase in the *Drosophila melanogaster* species subgroup (*D. melanogaster* and its closest relatives). Seventy-two sequences are from *D. melanogaster* itself, and 14 are from the other species. Among the 67 variable amino acids, 27 (numbered in red) are variable in *D. melanogaster*. The other species are boxed in red. « Prox » and « distal » refer to the two Amy gene copies.

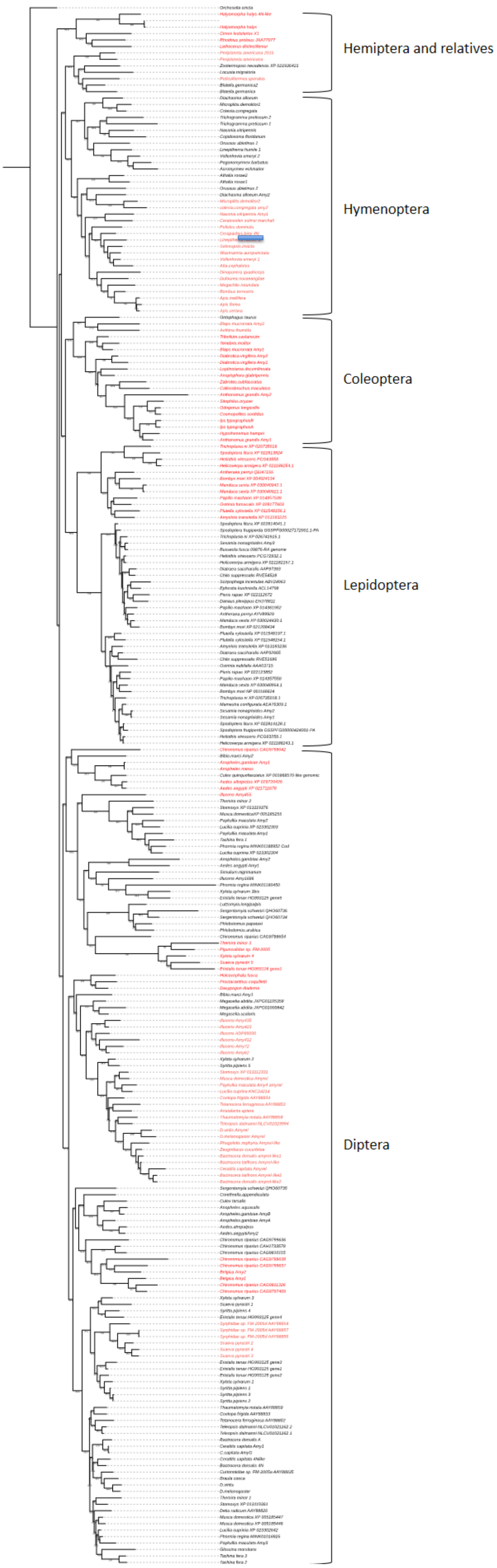

Supplementary Figure S14. Maximum-likelihood tree of 240 insect α-amylase sequences. Sequences devoid of the GHGA loop are labelled in red. The tree was rooted with the collembola *Orchesella cincta*.

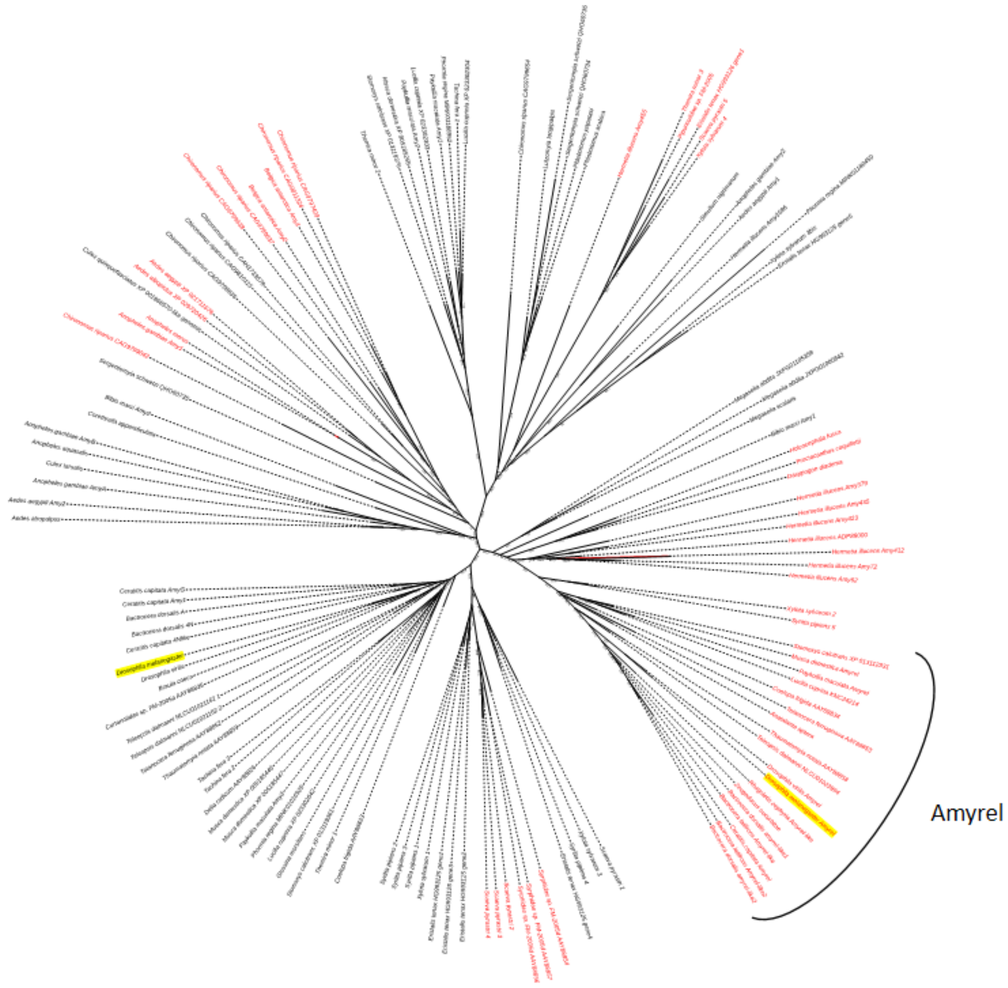

Supplementary Figure S15. Unrooted Maximum-likelihood tree of 127 α-amylase sequences representing 54 dipteran species. Sequences devoid of the GHGA loop are in labelled in red. The classical α-amylase, DMA, and the paralog Amyrel from *Drosophila melanogaster* are highlighted in yellow. The Amyrel cluster is indicated. Numbers along the branches are bootstrap values.

Supplementary Table S1. Ancestral state reconstruction at the sites considered as positively selected in the *D. melanogaster* branch (adapted from Araki et al. [67]).

Amino acid position (mature protein)	Replacement in *D. melanogaster* (Araki et al 2001)	Ancestral state reconstruction using marginal likelihood	Marginal probability	Probability associated with the mode (sampled reconstruction)
37	F->Y	Y	0.618	0.57
75	Q->E	E	0.997	1.00
138	N->S	T	0.990	0.99
145	Q->E	Q	0.553	0.61
333	S->T	T	0.640	0.72
351	V->I	I	0.648	0.69
448	S->T	T	0.559	0.507
